# A DFT study of uranyl hydroxyl complexes: structure and stability of trimers and tetramers

**DOI:** 10.1007/s10967-017-5305-z

**Published:** 2017-05-31

**Authors:** Ewelina Grabias, Marek Majdan

**Affiliations:** 1 Institute of Mathematics, Maria Curie Skłodowska University, pl. Marii Curie Skłodowskiej 1, 20-031 Lublin, Poland; 2 Faculty of Chemistry, Maria Curie Skłodowska University, pl. Marii Curie Skłodowskiej 2, 20-031 Lublin, Poland

**Keywords:** Uranium, U(VI) hydroxy complexes, DFT calculations, Bond length

## Abstract

A DFT study of U(VI) hydroxy complexes was performed with special attention paid to the [(UO_2_)_3_(OH)_5_(H_2_O)_4–7_]^+^ and [(UO_2_)_4_(OH)_7_(H_2_O)_5–8_]^+^ species. It was established that the ionicity of the U=O bond increased when moving from [(UO_2_)(H_2_O)_5_]^2+^, [(UO_2_)_2_(OH)(H_2_O)_8_]^3+^, [(UO_2_)_2_(OH)_2_(H_2_O)_6_]^2+^, [(UO_2_)_3_(OH)_5_(H_2_O)_4–6_]^+^ to [(UO_2_)_4_(OH)_7_(H_2_O)_5–8_]^+^ species. In both [(UO_2_)_3_(OH)_5_(H_2_O)_4–6_]^+^ and [(UO_2_)_4_(OH)_7_(H_2_O)_5–8_]^+^ complexes, the U=O bond was observed to have a range of different lengths which depended on the composition of the first coordination sphere of UO_2_^2+^. The cyclic structures of trimeric complexes were somewhat more stable than their linear structures, which was probably due to the steric effect.

**Electronic supplementary material:**

The online version of this article (doi:10.1007/s10967-017-5305-z) contains supplementary material, which is available to authorized users.

## Introduction

Investigation of uranium complexes is very important from the practical point of view. Uranium is a toxic and radioactive element and its elimination from aqueous solutions near nuclear power plants or from underground waters in the neighborhood of uranium mines is a matter of global concern. The key to successful removal of this element is detailed information concerning the stability and structure of aqueous uranium complexes. Such information allows one to predict the migration of U ions with the different oxidation numbers in soil and the aqueous environment [1, 2]. The best known U compounds are those with oxidation number VI, and among them U(VI) hydroxy complexes, which are very attractive due to the large variation in the number and properties of the species they form. In acidic solutions, U(VI) exists in the form of ionic monomers UO_2_(H_2_O)_5_^2+^ [3–6], whereas in neutral and alkaline solutions, a vast array of cationic and anionic species occur, in which the coordination number 4 or 5 is preserved for UO_2_^2+^ ions [7–10]. The best known complexes: UO_2_(OH)^+^, (UO_2_)_2_(OH)^3+^, (UO_2_)_2_(OH)_2_^2+^, (UO_2_)_3_(OH)_4_^2+^, (UO_2_)_3_(OH)_5_^+^, (UO_2_)_4_(OH)_7_^+^, UO_2_(OH)_2_, UO_2_(OH)_3_^−^, and UO_2_(OH)_4_^2−^ have been investigated in detail, using different methods such as UV–vis [11–13], luminescence [14–16], FTIR [11, 17], Raman [11, 18], EXAFS spectroscopy [7, 19], potentiometry [20], and calorimetry [21]. Parallel to the experimental investigations, computational techniques have been used to predict or confirm the structure of U(VI) hydroxy complexes. Different methods of quantum-chemical calculations can be employed in the analysis of the electronic structure and properties of 5-f elements. The Density Functional Theory (DFT) method can serve as a tool to complement experimental studies and confirm the information coming from experimental work. According to Schreckenbach and Shamov, who studied actinide elements, generalized gradient approximation (GGA) functionals provide accurate geometries and frequencies, while hybrid functionals are superior for energetics. Those authors have established that the best results for actinides can be obtained when the first coordination sphere is treated *explicitly* and the second one is represented using the continuum solvation models [22]. They found that the application of the *explicit* model for the second coordination sphere of the actinides not only brought no clear advantages, but also made the calculations more time-consuming.

The aim of our contribution was to analyze the structure of U(VI) hydroxy complexes using the DFT method, with special attention being paid to the trimeric complexes (UO_2_)_3_(OH)_5_^+^ (henceforth referred to as 3.5) and the tetrameric complexes (UO_2_)_4_(OH)_7_^+^ (henceforth referred to as 4.7). Detailed studies of dimeric and trimeric complexes performed by Tsushima et al. [7] have shown that cyclic trimers with an oxo bond are stable. We decided to elucidate the structure of the linear species, keeping in mind the fact that in the real conditions they probably exist parallel to the cyclic isomers. Tetrameric complexes were investigated for the first time. Their concentration in aqueous solutions in the pH range of 5–6 is comparable to that of trimeric complexes (Fig. S1) [23], which means that the knowledge of their structure would help specialists understand the numerous complex equilibria present in the aqueous phase during various industrial processes, such as ion-exchange, solvent extraction, adsorption, etc., used for the reprocessing of uranium minerals or for the recovery of U from nuclear wastes [24–27].

## Experimental

### Computational details

All calculations for systems of uranium complexes were carried out using the Amsterdam Density Functional package (ADF2016) [28–30] at the relativistic level of theory, where the scalar coupling effects were considered using a two-component Hamiltonian with the zeroth order regular approximation (ZORA) [31–33]. Triple-ζ Slater basis set with two polarization functions were used for uranium and triple-ζ Slater basis set with one polarization function were used for oxygen and hydrogen [34]. The frozen core approximation was applied, leaving the 5*f*, 6*s*, 6*p*, 6*d* and 7*s* electrons of uranium, and the 2*s* and 2*p* electrons of oxygen (as well as the 1*s* of H) for explicit treatment. All calculations were done using the PW91 generalized gradient approximation (GGA) functional proposed by Perdew and Wang [35]. All structures were optimized in the aqueous phase by means of the Conductor like Screening Model (COSMO) of solvation as implemented in ADF package [36] using the Solvent Accessible Surface (asurf) of the solvent cavity.

The structures of uranium complexes were fully optimized without any constraint (except for [UO_2_(H_2_O)_5_]^2+^, where a symmetry point group (D5h) was applied). A frequency analysis showed that all the investigated structures had only positive frequencies confirming that these were local minima on the energy surface. The Mayer bond order [37] and atomic charges were obtained from fully optimized electronic structures.

### FT-IR analysis

The FT-IR spectra of UO_2_(NO_3_)_2_ aqueous solutions were recorded in the transmission mode at room temperature on a 1725X Perkin Elmer instrument using the KBr pellet technique at 4 cm^−1^ resolution. The KBr was dried in a drier at 200 °C for 24 h. The tablets (radius 1 cm, thickness 0.1 cm) were prepared using a hydraulic press. The 560 mg KBr was mixed with UO_2_(NO_3_)_2_ aqueous solution. Uranyl nitrate solutions were prepared from UO_2_(NO_3_)_2_·6H_2_O (Lachema n.p. Brno, p.a.). The U(VI) spectra were deconvoluted with the second-derivative method using Peak-Fit software V.4 (SeasolveSoftware Inc.).

## Results and discussion

### Geometry of the U(VI) complexes

**Fig. 1:**
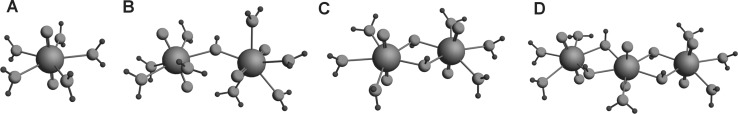
Geometries of uranyl **a** monomer (1.0); **b** dimer (2.1); **c** dimer (2.2); **d** trimer (3.4) complexes optimized in the aqueous phase

**Fig. 2:**
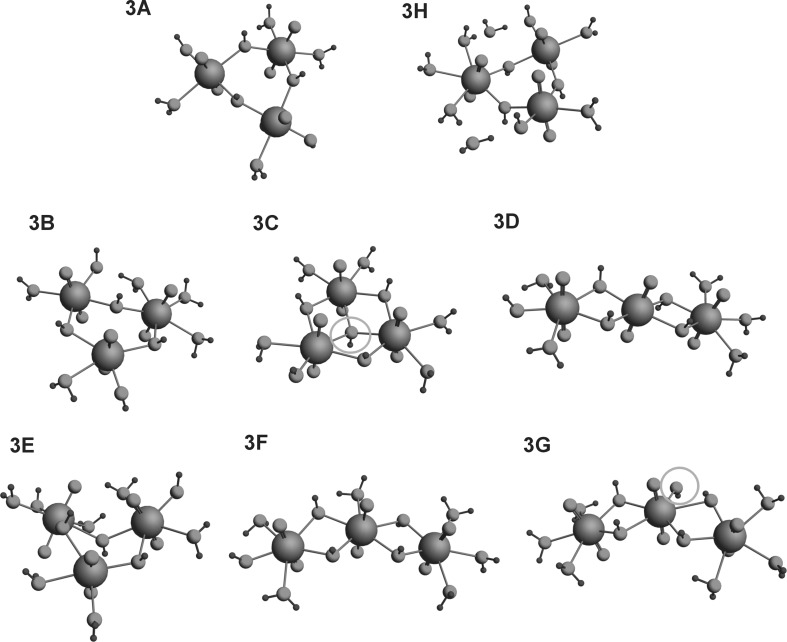
Geometries of uranyl trimeric hydroxy complexes (3.5) optimized in the aqueous phase

**Fig. 3:**
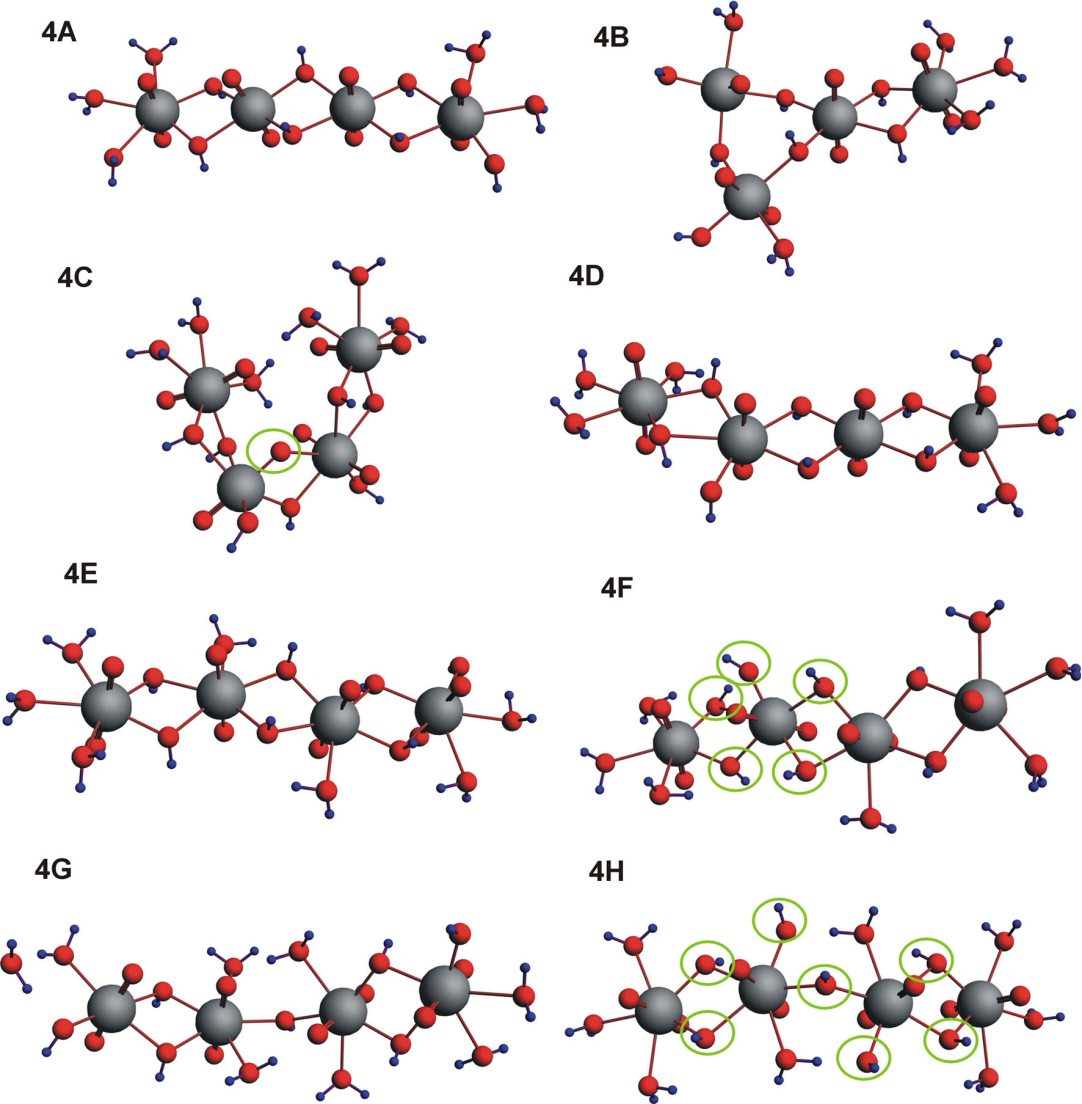
Geometries of uranyl tetrameric hydroxy complexes (4.7) optimized in the aqueous phase

The structures of all the investigated complexes are given in Figs. 1, 2 and 3. The following species were examined: UO_2_(H_2_O)_5_^2+^ (designated 1.0), (UO_2_)_2_(OH)^3+^ (2.1), (UO_2_)_2_(OH)_2_^2+^ (2.2), (UO_2_)_3_(OH)_4_^2+^ (3.4), (UO_2_)_3_(OH)_5_^+^ (3.5) and (UO_2_)_4_(OH)_7_^+^ (4.7). The parameters for the dimeric complexes (UO_2_)_2_(OH)^3+^ and (UO_2_)_2_(OH)_2_^2+^ (designated respectively 2.1 and 2.2), i.e. U–U distances of 4.445 and 3.851 Å and O=U=O bond angles of 176° and 173.2°, respectively, are very similar to the values found by Tsushima, i.e. U–U 4.390 and 3.875 Å and U=O=U 175° for the 2.2 complex [7]. A detailed comparison of the structures of isomeric trimeric and tetrameric hydroxy complexes with different numbers of H_2_O ligands is given in Table 1. For complex 3.5.5, the cyclic structures 3B and 3C are more stable than the linear ones, but the cyclic structure 3C with a tridentate OH bridge in the center is less stable than 3B, which does not have a bridge of this type. The differences in stability are probably a consequence of a tension among the U–OH bonds inside the structural ring resulting from electrostatic repulsion by the four hydroxyls situated in the vicinity. Generally, all cyclic structures are more extended in space than the linear ones, which means that their U–U distances are larger and the mutual repulsion of ligands is weaker, leading to greater stability. For example the U–U distances of 3.975, 4.447, 3.935 Å characteristic of the 3B complex are larger than the U–U distances of 3.778, 3.836 Å characterizing the 3D complex. In the case of complex 3.5.6, the U–U distances characteristic of the most stable species, 3E, are 3.927, 3.894, and 4.563 Å, whereas the distances calculated for 3F and 3G are 3.817, 3.865; 3.816, and 3.854 Å. 3G is the least stable of these complexes. Apart from having shorter U–U distances, it is also characterized by accumulation of the negative charge, originating from five hydroxyls, in the center of the structure. Complex 4.7.5 has two isomers. One of them (B) is less stable because it has an asymmetric structure with three uranyl ions with coordination number 4. The other isomer (A) has a symmetric form, and since it contains only two uranyls with coordination number 4, it is more coordinatively saturated. Two isomers of species 4.7.6 differ remarkably. One of them (C) has an oxo bridge in its structure and for this reason is much more stable than isomer D, which does not have this bridge. Between the two isomers of species 4.7.7, 4F is less stable because the water molecule in the center of the structure has been replaced by a hydroxyl which has increased the repulsion among the hydroxyls. For the same reason, isomer F of complex 4.7.8 is less stable than isomer G.

**Table 1:** Relative energy for stochiometric equivalent of uranium hydroxy complexes

Uranyl complexes stoichiometry (UO_2_)_*x*_(OH)_*y*_(H_2_O)_*z*_	Complex number	Energy difference (kJ/mol)	Average Mulliken population of O *p* orbitals in OH bridges	Wavenumber (cm^−1^)
3.5.4	3A	–	4.987	928
3.5.5	3B	0	4.962	945
	3C	18.4	4.959	923
	3D	23.7	4.916	939
3.5.6	3E	0	4.952	920
	3F	8	4.924	938
	3G	45.3	4.901	932
3.5.7	3H	–	4.995	940
4.7.5	4A	0	4.922	943
	4B	10.6	4.958	921
4.7.6	4C	0	4.887	898
	4D	42.5	4.913	929
4.7.7	4E	0	4.939	942
	4F	38.1	4.926	932
4.7.8	4G	0	4.951	925
	4H	55.3	4.932	929

**Fig. 4:**
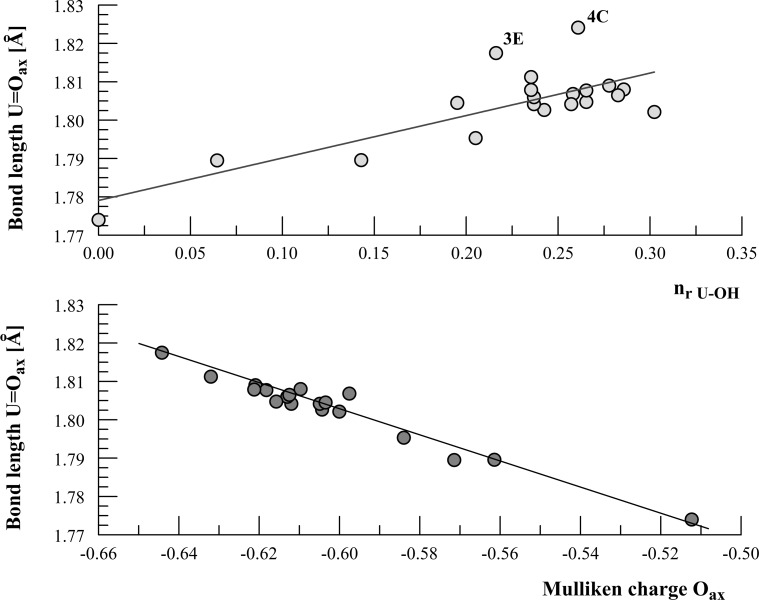
The changes in average U=O axial bond length in U(VI) hydroxy complexes

The changes in U=O bond length with the relative number n_r U–OH_ of U–OH bonds and with the average Mulliken charge of O axial atoms are given in Fig. 4. U=O bond length was calculated as the average length of all U=O bonds in a particular complex. The relative number of U–OH bonds *n*_r__U–OH_ = *n*_U–OH_/n, where *n*_r U–OH_ is the number of U–OH bonds and n denotes the sum of all bonds in the complex, i.e. the sum of U–OH, U=O, U–O, U–H_2_O, and O–H bonds. The length of U=O visibly increases with *n*_r U–OH_ and decreases with the charge of axial oxygens. For structures 3E and 4C, the lengthening of the bond is especially pronounced due to the steric effect in the case of 3E, i.e. the presence of two uranyls with the coordination number 5, and due to the lowering of the number of U=O bonds in 4C resulting from the formation of an oxo bridge. The phenomenon of U=O bond change is consistent with the reports of many authors who noticed that U=O bonds weakened when some electron donors were present in the equatorial plane of the U(VI) complex [4], which was explained as a result of a concurrent *π* donation from 2*p* orbitals of axial and equatorial O atoms to the 5*f* and 6*d* orbitals of U atoms. Worth mentioning are the results of calculations done by Tsushima et al. [3] and Fujii et al. [38], who analyzed UO_2_(OH)_2_ and (UO_2_)_2_(OH)_2_^2+^ species. The bend of the O=U=O bond is unquestionable and more pronounced for the latter species.

The increase of the Lewis acidity of the oxo uranyl ligand (U=O) under the influence of the different equatorial oxygen and nitrogen ligands may be exploited in the synthesis of the different adduct complexes in which the length of U=O bond changes in the range 1.8–1.9 Å [39]. This is very close to the change in U=O bond, which can be found in our work, i.e. 1.77–1.82 Å. The most interesting is the formation of the hydrogen bond between the uranyl oxygen (U=O) and amines [40, 41] and this fact potentially is important from the point of view of U(VI) extraction from the aqueous environment. Sather, Berryman and Rebek [42] showed that tripodal receptor with carboxylate and amide groups in its structure forming the hydrogen bonds with uranyl oxygens (U=O), is able to extract selectively uranium from the sea water. Uranyl hydrated oxides and hydroxides form different structures with hydrogen bonded water in the interlayer regions [43] which is important in uranium processing and extraction in various steps of the nuclear fuel cycle.

**Fig. 5:**
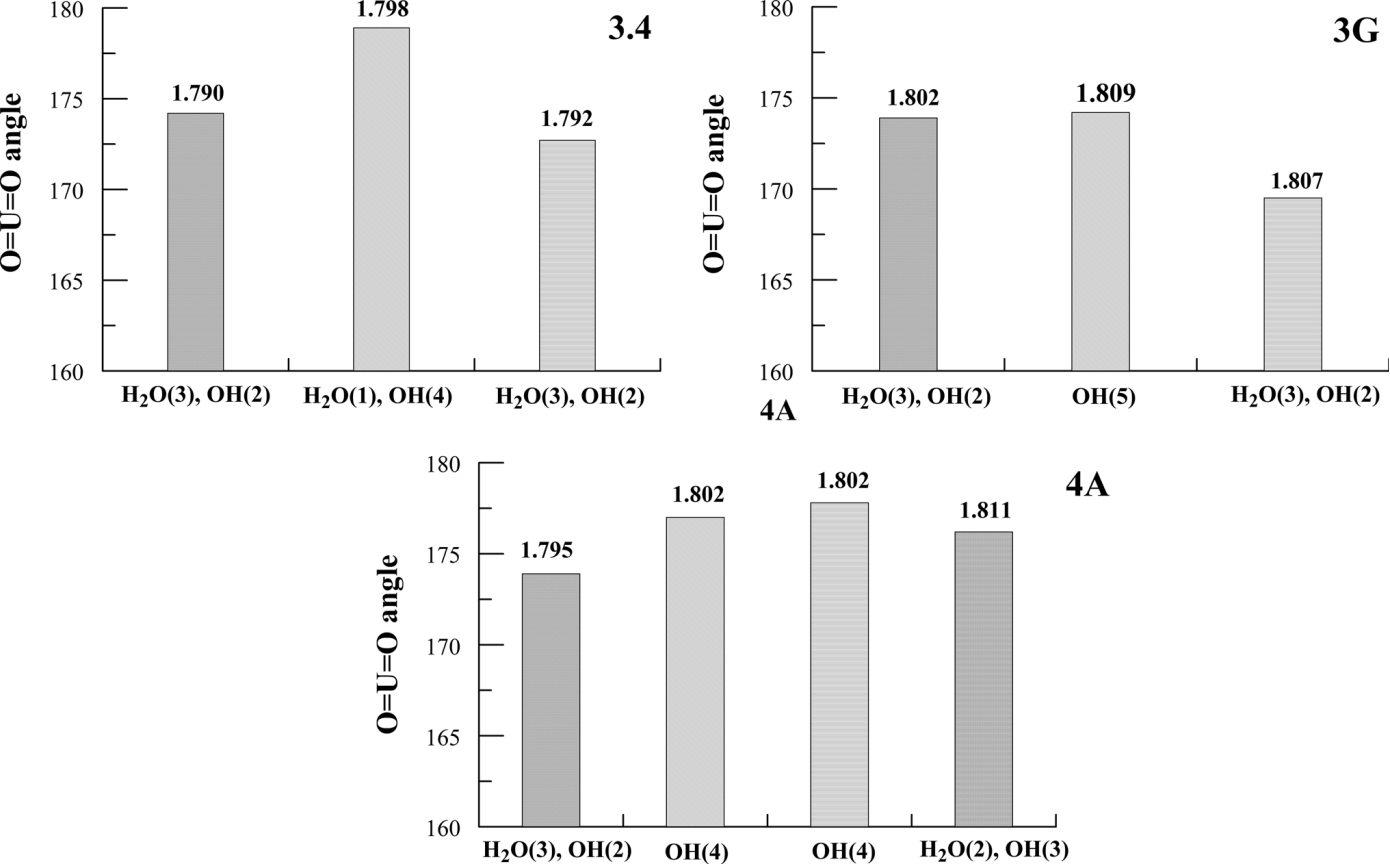
The changes in O=U=O average angle in 3.4, 3A and 4A U(VI) hydroxy complexes [numbers denotes U=O bond length (Å)]

According to the observations made in this present work, the change in O=U=O bond angle depends on the composition of the first coordination sphere of the UO_2_^2+^ ion, and this angle is evidently wider for higher numbers of hydroxyls coordinating uranyl ions (Fig. 5). When a uranyl ion is coordinated exclusively by hydroxyls, the average O=U=O bond angle is close to 180°, in contrast to peripheric UO_2_^2+^ ions in hydroxyl complexes of U(VI), in which this angle is visibly narrower. It seems that the *π* donation from 2*p* orbitals of axial O atoms is counterbalanced by the donation originating from the orbitals of the equatorial O atoms of hydroxyls. No such counterbalance is present when hydroxyl ligands are replaced by H_2_O ligands, which are weaker electron donors. The unsymmetrical and inhomogeneous environment of UO_2_^2+^ ions in the first coordination sphere results in the shortening of the U=O bond paralleled by the reduction of the O=U=O bond angle. This observation is true of species 3.4 and 3.5, but does not apply to species 4.7. Although the average O=U=O angle for the peripheric uranium atoms is narrower than for the atoms in the center of the structure, the change in U=O bond length contradicts the above mentioned observation. For example for species 4A (4.7.5) the angles are: 173.9°, 177°, 177.8°, 176.2°, but the lengths of the U=O bonds form an unexpected sequence: 1.795, 1.802, 1.802, 1.811 Å, with a high value for the peripheric U atom which suggests that bond length is not a simple function of *π* donation from 2*p* orbitals of axial oxygen.

In our opinion, the formation of higher hydroxy complexes of U(VI) in moving from monomers to dimers to trimers to tetramers results in a spatial expansion of the species formed, which impedes the overlapping of 5*f* and 6*d* uranium orbitals with 2*p* orbitals of oxygen and increases the ionicity of U=O bonds by increasing the number of hydroxyl ligands in the first coordination sphere of UO_2_^2+^.

**Fig. 6:**
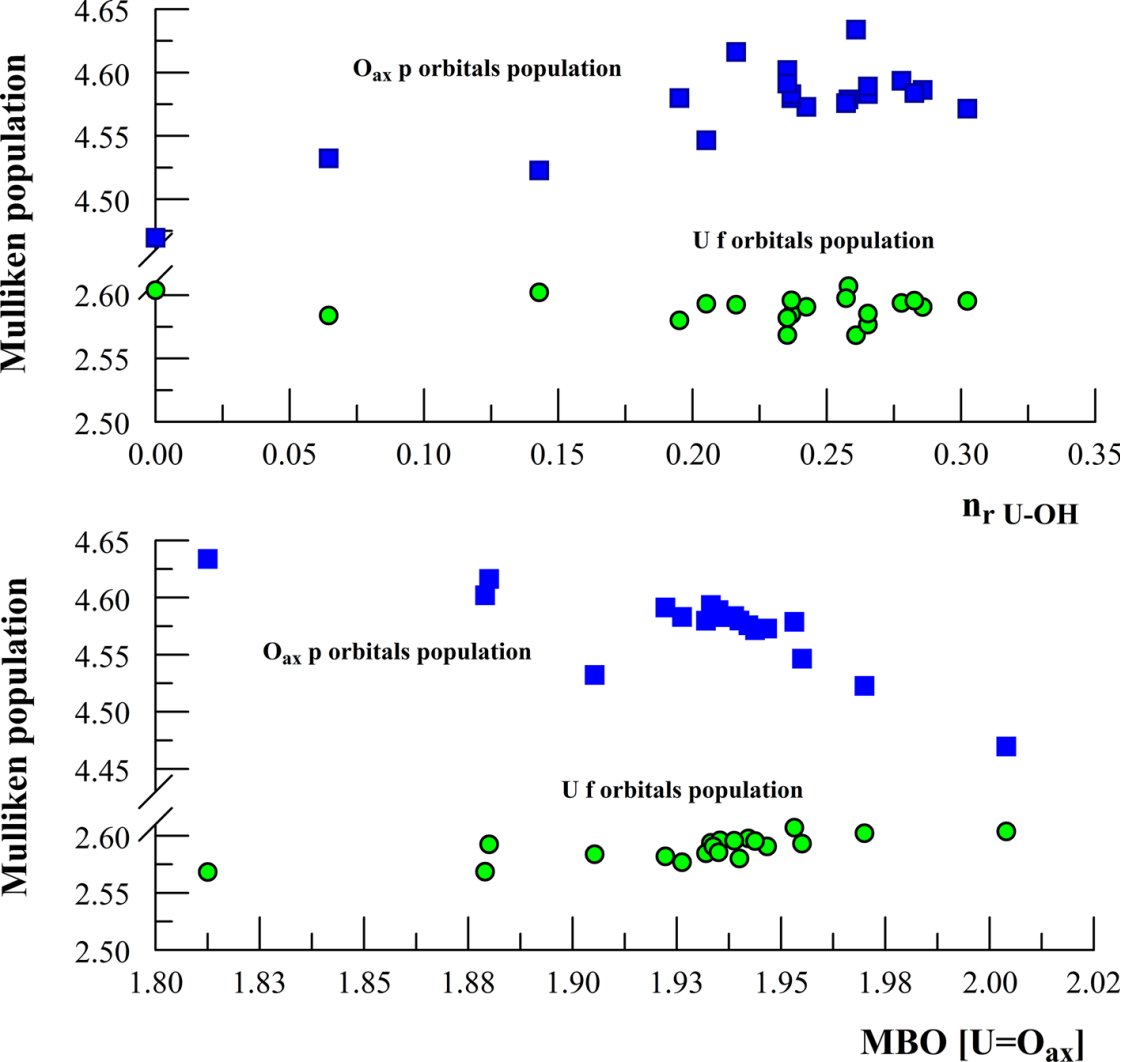
The changes in average Mulliken population of the U 5*f* and O axial 2*p* orbitals

The changes in average Mulliken population for 5*f* and 2*p* orbitals of U and axial O versus *n*_r__U–OH_ and Mayer bond order (MBO) of U=O bond, respectively, are shown in Fig. 6. The average Mulliken population of 2*p* orbitals increases with the increasing *n*_r__U–OH_ and with the decreasing Mayer bond order (MBO); this is in contrast to the population of 5*f*, which decreases with n_r__U-OH_, providing evidence for some cancellation of *f*–*p* overlapping. The Mulliken populations for O in OH bridges (for trimeric and tetrameric species) are given in Table 1. It is easy to notice that for the cyclic structures 3B, 3C, and 3E, the Mulliken populations are higher than for the linear structures 3D, 3F, and 3G. A similar situation holds for structures 4B and 4A; this indicates that bridged U–OH bonds are more ionic in the cyclic structures than in the linear ones.

### Molecular orbital analysis

The HOMO–LUMO diagrams for isomeric trimers (UO_2_)_3_(OH)_5_^+^ and tetramers (UO_2_)_4_(OH)_7_^+^ are given in Fig. S2. Molecular orbital composition and energies are included in Table 2. The molecular orbitals HOMO (the Highest Occupied Molecular Orbital) are localized on O 2*p* orbitals originating from U=O, H_2_O, OH^−^ and on *p*, *f* orbitals from U. The participation of *p* orbitals from OH^−^ and H_2_O ligands in the overall energy of HOMO orbital is the highest. The energy gap for 3B, 3C cyclic trimers is higher in comparison with 3D linear trimer. The same, the sequence 3E(cyclic) > 3F, 3G (linear) is preserved, therefore, one can conclude that cyclic trimeric isomers are more stable than the linear ones. For the tetrameric complexes we have found the following sequences concerning HOMO–LUMO energy gap: 4A > 4B; 4C > 4D; 4E < 4F; 4G > 4H, which is the same as the sequence of the relative total bond energies of particular species, except for 4E/4F species pair. The LUMO (the Lowest Unoccupied Molecular Orbital) orbitals are localized mainly on U *f* orbitals for all analyzed trimeric and tetrameric structures. Generally, the energy gap for tetrameric complexes is lower than for the trimeric ones and for this reason one can presume that tetramers (UO_2_)_4_ (OH)_7_^+^ would show more chemical reactivity.

**Table 2:** Molecular orbital composition of uranyl hydroxy trimeric and tetrameric complexes

Complex number	*E*_HOMO_ (eV)	*E*_LUMO_ (eV)	Δ*E*	Molecular orbitals contributions (%)	
				HOMO	LUMO
3A	−7.267	−5.768	1.499	23.88 ($f_{z^{3}}$, $f_{z^{2}y}$, *f*_*x*_, *f*_*y*_, *p*_*y*_, *p*_*z*_) U 22.75 (*p*_*x*_, *p*_*y*_, *p*_*z*_) O (U=O) 43.03 (*p*_*x*_, *p*_*y*_, *p*_*z*_) O (H_2_O/OH)	97.83 ($f_{z^{3}}$, $f_{z^{2}x}$, $f_{z^{2}y}$, *f*_*xyz*_, *f*_*x*_, *f*_*y*_) U
3B	−7.378	−5.653	1.725	28.30 ($f_{z^{3}}$, $f_{z^{2}x}$, $f_{z^{2}y}$, *f*_*x*_, *f*_*y*_, *f*_*z*_, *p*_*y*_, *p*_*z*_) U 23.61 (*p*_*x*_, *p*_*y*_, *p*_*z*_) O (U=O) 41.96 (*p*_*x*_, *p*_*y*_, *p*_*z*_) O (H_2_O/OH)	97.14 ($f_{z^{3}}$, $f_{z^{2}x}$, *f*_*xyz*_, *f*_*y*_, *p*_*x*_) U
3C	−7.243	−5.305	1.938	28.35 ($f_{z^{3}}$, $f_{z^{2}x}$, $f_{z^{2}y}$, *f*_*x*_, *f*_*z*_, *p*_*z*_) U 24.38 (*p*_*y*_, *p*_*z*_) O (U=O) 38.61 (*p*_*x*_, *p*_*y*_, *p*_*z*_) O (H_2_O/OH)	94.88 ($f_{z^{2}y}$, $f_{z^{2}x}$, *f*_*x*_, *f*_*y*_, *f*_*z*_) U
3D	−7.268	−5.776	1.492	25.48 ($f_{z^{3}}$, $f_{z^{2}x}$, $f_{z^{2}y}$, *f*_*xyz*_, *f*_*x*_, *f*_*z*_, *p*_*x*_, *p*_*z*_) U 23.85 (*p*_*x*_, *p*_*y*_, *p*_*z*_) O (U=O) 42.24 (*p*_*x*_, *p*_*y*_, *p*_*z*_) O (H_2_O/OH)	96.35 ($f_{z^{3}}$, $f_{z^{2}x}$, $f_{z^{2}y}$, *f*_*x*_, *f*_*z*_) U
3E	−7.527	−5.301	2.226	25.32 ($f_{z^{3}}$, $f_{z^{2}x}$, $f_{z^{2}y}$, *f*_*xyz*_, *f*_*y*_, *p*_*y*_, *p*_*z*_) U 22.58 (*p*_*x*_, *p*_*y*_, *p*_*z*_) O (U=O) 41.98 (*p*_*x*_, *p*_*y*_, *p*_*z*_) O (H_2_O/OH)	95.77 ($f_{z^{3}}$, $f_{z^{2}x}$, *f*_*xyz*_, *f*_*x*_, *f*_*y*_, *f*_*z*_) U
3F	−7.315	−5.555	1.760	21.64 ($f_{z^{3}}$, $f_{z^{2}x}$, $f_{z^{2}y}$, *f*_*xyz*_, *f*_*x*_, *f*_*y*_, *p*_*x*_, *p*_*z*_) U 21.44 (*p*_*x*_, *p*_*z*_) O (U=O) 46.27 (*p*_*x*_, *p*_*z*_) O (H_2_O/OH)	96.19 ($f_{z^{3}}$, $f_{z^{2}x}$, $f_{z^{2}y}$, *f*_*xyz*_, *f*_*z*_) U
3G	−7.080	−5.333	1.747	21.19 ($f_{z^{3}}$, $f_{z^{2}x}$, *f*_*xyz*_, *f*_*x*_, *p*_*y*_, *p*_*z*_) U 22.17 (*p*_*y*_, *p*_*z*_) O (U=O) 40.55 (*p*_*y*_, *p*_*z*_) O (H_2_O/OH)	96.11 ($f_{z^{3}}$, $f_{z^{2}x}$, *f*_*x*_, *f*_*y*_, *f*_*z*_) U
3H	−7.615	−5.301	2.314	33.01 ($f_{z^{3}}$, *f*_*xyz*_, $f_{z^{2}y}$, $f_{z^{2}x}$, *f*_*y*_, *f*_*z*_, *p*_*z*_) U 24.45 (*p*_*x*_, *p*_*y*_, *p*_*z*_) O (U=O) 32.38 (*p*_*x*_, *p*_*y*_, *p*_*z*_) O (H_2_O/OH)	97.28 ($f_{z^{2}x}$, $f_{z^{2}y}$, $f_{z^{3}}$, *f*_*xyz*_, *f*_*x*_, *f*_*y*_) U
4A	−7.211	−5.942	1.269	25.08 ($f_{z^{3}}$, $f_{z^{2}y}$, $f_{z^{2}x}$, *p*_*x*_, *p*_*z*_) U 23.42 (*p*_*y*_, *p*_*z*_) O (U=O) 40.21 (*p*_*y*_, *p*_*z*_) O (H_2_O/OH)	95.56 ($f_{z^{2}y}$, $f_{z^{3}}$, *f*_*xyz*_, *f*_*y*_, *f*_*z*_) U
4B	−7.005	−5.799	1.206	21.9 ($f_{z^{3}}$, $f_{z^{2}y}$, $f_{z^{2}x}$, *p*_*x*_, *p*_*z*_) U 22.64 (*p*_*x*_, *p*_*y*_, *p*_*z*_) O (U=O) 42.54 (*p*_*x*_, *p*_*y*_, *p*_*z*_) O (H_2_O/OH)	96.56 ($f_{z^{2}x}$, $f_{z^{2}y}$, *f*_*xyz*_, *f*_*x*_, *f*_*y*_, *f*_*z*_) U
4C	−7.066	−5.573	1.493	19.41 (*f*_*xyz*_, $f_{z^{2}x}$, *f*_*y*_, *f*_*z*_, *p*_*x*_, *p*_*y*_) U 22.14 (*p*_*x*_, *p*_*y*_, *p*_*z*_) O (U=O) 44.08 (*p*_*x*_, *p*_*y*_, *p*_*z*_) O (H_2_O/OH)	95.02 ($f_{z^{2}x}$, $f_{z^{2}y}$, *f*_*xyz*_, *f*_*y*_, *f*_*z*_) U
4D	−6.970	−5.696	1.274	25.78 (*f*_*xyz*_, $f_{z^{2}x}$, $f_{z^{2}y}$, *f*_*x*_, *f*_*z*_, *p*_*z*_) U 23.71 (*p*_*x*_, *p*_*y*_, *p*_*z*_) O (U=O) 39.47 (*p*_*y*_, *p*_*z*_) O (H_2_O/OH)	96.24 ($f_{z^{2}x}$, $f_{z^{2}y}$, $f_{z^{3}}$, *f*_*xyz*_, *f*_*x*_, *f*_*y*_, *f*_*z*_) U
4E	−7.044	−5.664	1.380	30.99 ($f_{z^{3}}$, $f_{z^{2}x}$, $f_{z^{2}y}$, *f*_*x*_, *f*_*z*_, *p*_*y*_, *p*_*z*_) U 24.58 (*p*_*y*_, *p*_*z*_) O (U=O) 38.95 (*p*_*x*_,*p*_*y*_, *p*_*z*_) O (H_2_O/OH)	97.08 ($f_{z^{2}x}$, $f_{z^{2}y}$, *f*_*xyz*_, *f*_*x*_, *f*_*y*_, *f*_*z*_) U
4F	−6.987	−5.548	1.439	23.34 ($f_{z^{3}}$, $f_{z^{2}x}$, $f_{z^{2}y}$, *f*_*xyz*_ *f*_*x*_, *p*_*z*_) U 19.12 (*p*_*y*_, *p*_*z*_) O (U=O) 46.81 (*p*_*x*_,*p*_*y*_, *p*_*z*_) O (H_2_O/OH)	96.42 ($f_{z^{3}}$, $f_{z^{2}x}$, $f_{z^{2}y}$, *f*_*xyz*_, *f*_*x*_, *f*_*y*_, *f*_*z*_) U
4G	−7.424	−5.564	1.860	26.21 ($f_{z^{3}}$, $f_{z^{2}x}$, $f_{z^{2}y}$, *f*_*xyz*_ *f*_*y*_, *f*_*z*_, *p*_*y*_, *p*_*z*_) U 23.49 (*p*_*y*_, *p*_*z*_) O (U=O) 41.61 (*p*_*y*_, *p*_*z*_) O (H_2_O/OH)	94.10 ($f_{z^{2}y}$, *f*_*xyz*_, *f*_*x*_, *f*_*y*_, *f*_*z*_) U
4H	−6.912	−5.145	1.767	16.81 ($f_{z^{2}y}$, *f*_*xyz*_ *f*_*x*_, *f*_*z*_, *p*_*y*_, *p*_*z*_) U 17.84 (*p*_*x*_, *p*_*y*_, *p*_*z*_) O (U=O) 52.59 (*p*_*x*_, *p*_*y*_, *p*_*z*_) O (H_2_O/OH)	96.31 ($f_{z^{3}}$, $f_{z^{2}x}$, $f_{z^{2}y}$, *f*_*xyz*_, *f*_*x*_, *f*_*y*_, *f*_*z*_) U

### FT-IR spectra of UO_2_(NO_3_)_2_ solutions

**Fig. 7:**
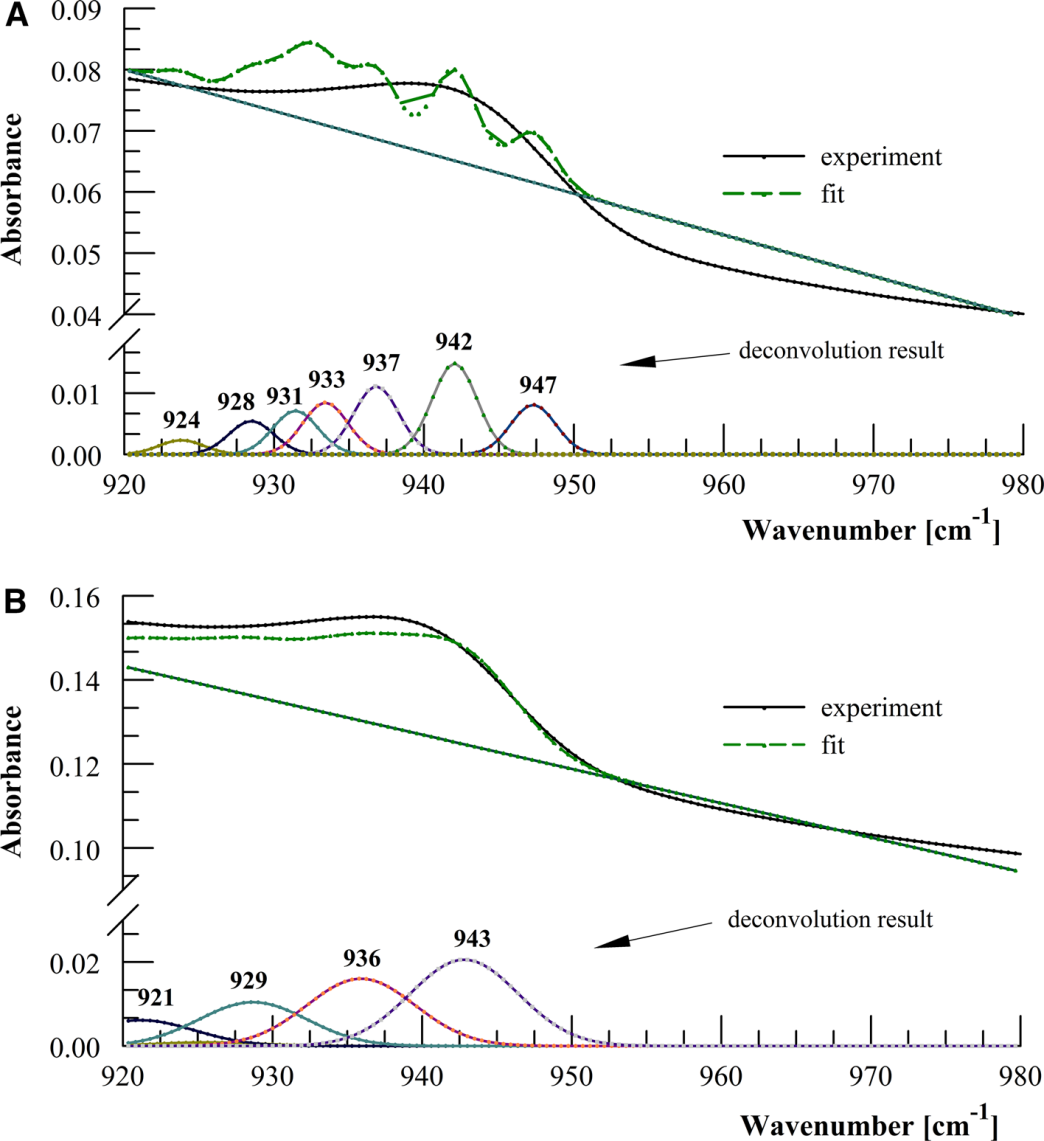
FTIR spectra of UO_2_(NO_3_)_2_ aqueous solutions (0.05 mol L^−1^) **a** pH 4.92, **b** pH 5.23

The deconvoluted FT-IR spectra of UO_2_(NO_3_)_2_ solutions with different pH values, characteristic of the predominance of 3.5 and 4.7 complexes in the U(VI) solution, are given in Fig. 7. The bands in the range 920–980 cm^−1^ are characteristic of different 3.5 and 4.7 species (Table 1), i.e. of the asymmetric O=U=O vibrations, however, it is very difficult to unequivocally determine whether they belong to trimeric or tetrameric species because trimers and tetramers have very similar U=O bond energies. One has to take into account the results obtained by Muller et al. [17], who found characteristic frequencies of 923 and 940 cm^−1^ for (UO_2_)_3_(OH)_5_^+^ and (UO_2_)_2_(OH)_2_ species, respectively. It is difficult to imagine that the frequencies 942, 947, and 943 cm^−1^ observed in our spectra could originate from a dimer, since only very small numbers of dimers are present in a U(VI) solution at pH range 5–6 (Fig. S1).

## Conclusions

The length of a U=O bond increases with the number of hydroxyl ligands in the first coordination sphere of UO_2_^2+^ ions, parallel to a decrease in the Mayer bond order and an increase in the Mulliken population on O 2*p* orbitals and a slight drop in this parameter on 5*f* U orbitals. The strengthening of the ionic character of the U=O bond, when it passes from UO_2_^2+^ to the (UO_2_)_4_(OH)_7_^+^ complex, is therefore unquestionable.

The O=U=O bond angle is wider for uranyls coordinated exclusively by hydroxyl ligands compared with those which have a mixture of H_2_O and OH^−^ ligands in the first coordination sphere.

For complex 3.5.5, the cyclic structures are more stable than the linear ones, which probably is the consequence of the steric effect, i.e. weaker repulsion of ligands in the first coordination sphere of the cyclic hydroxy complexes.

The values of U=O bond energy in (UO_2_)_3_(OH)_5_^+^ and (UO_2_)_4_(OH)_7_^+^ is very similar, which results in similar frequencies of O=U=O asymmetric stretching vibrations.

The ionic character of the bridged U–OH bond is stronger in cyclic structures 3.5.5 and 3.5.6 than in their linear counterparts. Further studies are necessary to support this conclusion and to extend it to species 4.7.5, 4.7.6, 4.7.7, 4.7.8.

The value of the energy gap of analyzed complexes suggests that cyclic trimeric isomers are more stable than the linear ones. HOMO orbitals are localized on O *p* and U *p*, *f* orbitals. LUMO orbitals are localized mainly on U *f* orbitals.

## Electronic supplementary material

Below is the link to the electronic supplementary material.

Supplementary material 1 (DOCX 942 kb)

## References

[1] Jezierski G (2005). Energia jądrowa wczoraj i dziś.

[2] Gavrilescu M, Pavel LV, Cretescu I (2009). Characterization and remediation of soils contaminated with uranium. J Hazard Mater.

[3] Tsushima S, Reich T (2001). A theoretical study of uranyl hydroxide monomeric and dimeric complexes. Chem Phys Lett.

[4] Ingram KIM, Haller LJL, Kaltsoyannis N (2006). Density functional theory investigation of the geometric and electronic structures of [UO_2_(H_2_O)_m_(OH)_n_]^2−n^ (n + m = 5). Dalton Trans.

[5] Weng Z, Wang S, Ling J, Morrison JM, Burns PB (2012). (UO_2_)_2_[UO_4_(trz)_2_](OH)_2_: a U(VI) coordination intermediate between a tetraoxido core and a uranyl ion with cation–cation interactions. Inorg Chem.

[6] Odoh SO, Schreckenbach G (2013). DFT study of uranyl peroxo complexes with H_2_O, F^−^, OH^−^, CO_3_^2−^, and NO_3_^−^. Inorg Chem.

[7] Tsushima S, Rossberg A, Ikeda A, Muller K, Scheinost AC (2007). Stoichiometry and structure of uranyl(VI) hydroxo dimer and trimer complexes in aqueous solution. Inorg Chem.

[8] Finch RJ, Cooper MA, Hawthorne FC, Ewing RC (1996). The crystal structure f schoepite. [(UO_2_)_8_O_2_(OH)_12_](H_2_O)_12_. Can Miner.

[9] Moll H, Rossberg A, Steudtner R, Drobot B, Müller K (2014). Tsushima S (2014) Uranium(VI) chemistry in strong alkaline solution: speciation and oxygen exchange mechanism. Inorg Chem.

[10] Odoh SO, Schreckenbach G (2013). DFT study of oxo-functionalized pentavalent dioxouranium complexes: structure, bonding, ligand exchange, dimerization, and U(V)/U(IV) reduction of OUOH and OUOSiH_3_ complexes. Inorg Chem.

[11] Quiles F, Nguyen-Trung C, Carteret C, Humbert B (2011). Hydrolysis of uranyl(VI) in acidic and basic aqueous solutions using a noncomplexing organic base: a multivariate spectroscopic and statistical study. Inorg Chem.

[12] Priyadarshini N, Sampath M, Kumar S, Mudali UK, Natarajan R (2013). A combined spectroscopic and light scattering study of hydrolysis of uranium (VI) leading to colloid formation in aqueous solutions. J Radioanal Nucl Chem.

[13] Meinrath G (1998). Chemometric analysis: uranium (VI) hydrolysis by UV-Vis spectroscopy. J. Alloys Compd.

[14] Eliet V, Grenthe I, Bidoglio G (2000). Time-resolved laser-induced fluorescence of uranium(VI) hydroxo-complexes at different temperatures. Appl Spectrosc.

[15] Meinrath G, Lis S, Stryla Z, Noubactep C (2000). Lifetime and fluorescence quantum yield of uranium(VI) species in hydrolyzed solutions. J Alloys Compd.

[16] Drobot B, Steudtner R, Raff J, Geipel G, Brendlera V, Tsushima S (2015). Combining luminescence spectroscopy, parallel factor analysis and quantum chemistry to reveal metal speciation—a case study of uranyl(VI) hydrolysis. Chem Sci.

[17] Muller K, Brendler V, Foerstendorf H (2008). aqueous uranium(VI) hydrolysis species characterized by attenuated total reflection Fourier-transform infrared spectroscopy. Inorg Chem.

[18] Quiles F, Burneau A (2000). Infrared and Raman spectra of uranyl VI oxo-hydroxo complexes in acid aqueous solutions: a chemometric study. Vib Spectrosc.

[19] Walshe A, Prüßmann T, Vitova T, Baker RJ (2014). An EXAFS and HR-XANES study of the uranyl peroxides [UO_2_(η_2_-O_2_)(H_2_O)_2_]·nH_2_O (n = 0, 2) and uranyl (oxy)hydroxide [(UO_2_)_4_O(OH)_6_]·6H_2_O. Dalton Trans.

[20] Palmer DA, Nguyen-Trung C (1995). Aqueous uranyl complexes. 3. Potentiometric measurements of the hydrolysis of uranyl(VI) ion at 25 °C. J Solut Chem.

[21] Zanonato PL, Di Bernardo P, Grenthe I (2014). Calorimetric study of the hydrolysis and peroxide complex formation of the uranyl(VI) ion. Dalton Trans.

[22] Schreckenbach G, Shamov GA (2010). Theoretical actinide molecular science. Acc Chem Res.

[23] Puigdomenech I (2010) MEDUSA—Make Equilibrium Diagrams Using Sophisticated Algorithms. http://www.kemi.kth.se/medusa/

[24] Ye X, Cui S, de Almeida V, Khomami B (2009). Interfacial complex formation in uranyl extraction by tributyl phosphate in dodecane diluent: a molecular dynamics study. J Phys Chem B.

[25] Johnson BE, Santschi PH, Chuang CY, Otosaka S, Addleman RS, Douglas M, Rutledge RD, Chouyyok W, Davidson JD, Fryxell GE, Schwantes JM (2012). Collection of lanthanides and actinides from natural waters with conventional and nanoporous sorbents. Environ Sci Technol.

[26] Manos MJ, Kanatzidis MG (2012). Layered metal sulfides capture uranium from seawater. J Am Chem Soc.

[27] Semnani F, Asadi Z, Samadfam M, Sepehrian H (2012). Uranium(VI) sorption behavior onto amberlite CG-400 anion exchange resin: effects of pH, contact time, temperature and presence of phosphate. Ann Nucl Energy.

[28] Fonseca Guerra C, Snijders JG, te Velde G, Baerends EJ (1998). Towards an order-N DFT method. Theor Chem Acc.

[29] te Velde G, Bickelhaupt FM, Baerends EJ, Fonseca Guerra C, van Gisbergen SJA, Snijders JG, Ziegler T (2001). Chemistry with ADF. J Comput Chem.

[30] ADF2016, SCM, Theoretical Chemistry, Vrije Universiteit, Amsterdam, The Netherlands, http://www.scm.com

[31] van Lenthe E, Baerends EJ, Snijders JG (1993). Relativistic regular two-component Hamiltonians. J Chem Phys.

[32] van Lenthe E, Baerends EJ, Snijders JG (1994). Relativistic total energy using regular approximations. J. Chem. Phys..

[33] van Lenthe E, Ehlers AE, Baerends EJ (1999). Geometry optimizations in the zero order regular approximation for relativistic effects. J Chem Phys.

[34] van Lenthe E, Baerends EJ (2003). Optimized Slater-type basis sets for the elements 1–118. J Comput Chem.

[35] Perdew JP, Wang Y (1992). Accurate and simple analytic representation of the electron-gas correlation energy. Phys Rev B.

[36] Pye CC, Ziegler T (1999). An implementation of the conductor-like screening model of solvation within the Amsterdam density functional package. Theor Chem Acc.

[37] Mayer I (1983). Charge, bond order and valence in the AB initio SCF theory. Chem Phys Lett.

[38] Fujii T, Fujiwara K, Yamana H, Moriyama H (2001). Raman spectroscopic determination of formation constant of uranyl hydrolysis species (UO_2_)_2_(OH)_2_^2+^. J Alloys Compd.

[39] Franczyk TS, Czerwinski KR, Raymond KN (1992). Stereognostic coordination chemistry. 1. The design and synthesis of chelators for the uranyl ion. J Am Chem Soc.

[40] Walton PH, Raymond KN (1995). Stereognostic coordination chemistry. 4. The design and synthesis of a selective uranyl ion complexant. Inorg Chim Acta.

[41] Sather AC, Berryman OB, Rebek J (2010). Selective recognition and extraction of the uranyl ion. J Am Chem Soc.

[42] Fortier S, Hayton TW (2010). Oxo ligand functionalization in the uranyl ion (UO_2_^2+^). Coord Chem Rev.

[43] Alam TM, Liao Z, Nyman M, Yates J (2016). Insight into hydrogen bonding of uranyl hydroxide layers and capsules by use of 1H magic-angle spinning NMR spectroscopy. J Phys Chem C.

