# Supplementary material for: A DFT study of uranyl hydroxyl complexes: structure and stability of trimers and tetramers
Source: J Radioanal Nucl Chem. 2017 May 31;313(2):455–65. doi: 10.1007/s10967-017-5305-z (PMC5533873; doi:10.1007/s10967-017-5305-z)
Supplement: Supplementary file 1 — Supplementary material 1 (DOCX 942 kb) [file 10967_2017_5305_MOESM1_ESM.docx]

Supplementary information


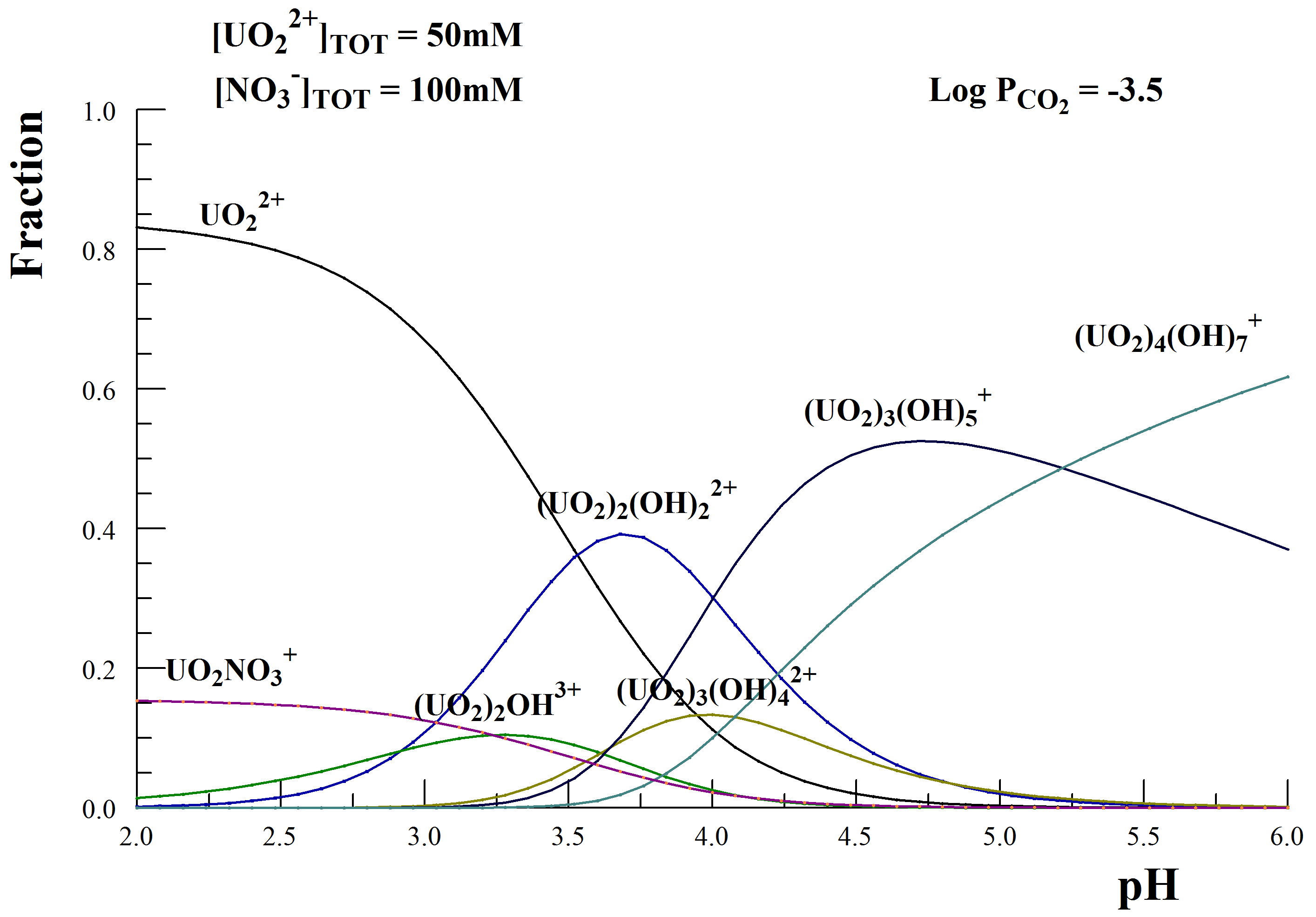


**Figure S1.** Uranium(VI) speciation in 0.05 mol L^-1^ UO_2_(NO_3_)_2_ aqueous solution based on Medusa software [16].


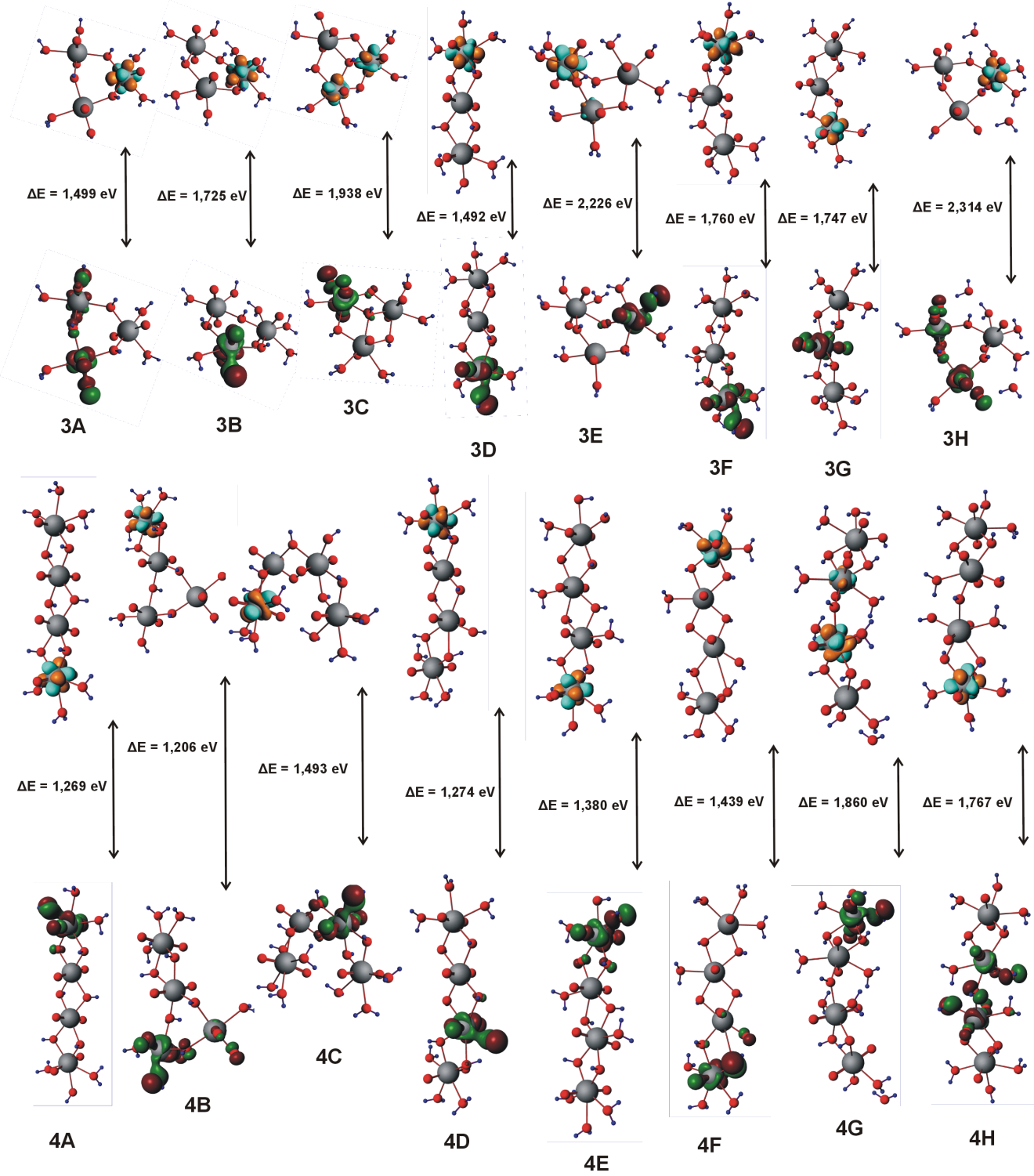


**Figure S2.** Frontier Molecular Orbitals of uranyl hydroxy trimers and tetramers.

Cartesian coordinates (Å) for the species under study calculated by Amsterdam Density Functional package (ADF2016).

1. [(UO_2_)(H_2_O)_5_] ^2+^

U -0.00000000 -0.00000000 0.00000000

H 2.47521570 1.79834361 -0.78338248

O -0.00000000 -0.00000000 -1.77408602

H -0.94545171 -2.90978582 0.78338248

O -0.00000000 -0.00000000 1.77408602

H -3.05953141 0.00000474 0.78338248

H 2.47521012 -1.79835128 -0.78338248

H -0.94544269 2.90978875 0.78338248

O 2.00289019 1.45518016 0.00000000

H 2.47521570 1.79834361 0.78338248

O 2.00288568 -1.45518637 0.00000000

H 2.47521012 -1.79835128 0.78338248

O -0.76503877 -2.35453479 0.00000000

H -0.94545171 -2.90978582 -0.78338248

O -2.47570564 0.00000384 0.00000000

H -3.05953141 0.00000474 -0.78338248

O -0.76503146 2.35453717 0.00000000

H -0.94544269 2.90978875 -0.78338248

(2.1) [(UO_2_)_2_(OH)(H_2_O)_8_]^3+^

H 10.46013201 -3.64940589 1.28404022

H 9.56299513 -4.89839971 1.58030666

H 7.21543707 -2.38391005 2.73170098

O 7.53386072 -2.03893784 1.87511051

H 3.89731417 0.03495588 -2.18625966

H 7.96219690 -1.18074689 2.06328666

O 1.59346193 -4.61511686 -0.84911949

U 7.73449746 -3.17058741 -0.31902440

U 3.34059179 -2.88020952 -0.92278163

H 0.80886781 -1.64051965 -2.38804470

H 5.91568509 -3.67547590 -2.80849225

O 1.64897262 -2.10946789 -2.55975262

O 9.60487652 -4.06850200 1.06635663

H 3.26962903 0.25276898 -0.77349913

H 5.62225143 -1.29178872 -0.37992357

H 1.53747957 -5.41909404 -1.40285403

H 7.39264877 -3.90266715 -3.34548734

O 8.54417881 -1.64122420 -0.72821764

O 2.48738698 -2.18791860 0.47268274

H 1.60914898 -2.42013659 -3.48583311

O 6.86913755 -3.52455463 -2.61371210

O 3.74355652 -0.40217484 -1.32484174

O 4.07355669 -3.63515894 -2.38142949

O 5.59951433 -2.27309372 -0.40243450

H 0.79064214 -4.59917971 -0.29152431

O 6.93612566 -4.70447715 0.19879287

O 4.20206053 -4.67287051 0.49160510

H 5.15147991 -4.95298207 0.48002282

H 3.79966249 -5.00897231 1.31562618

O 9.46783563 -4.09685698 -1.90290158

H 10.17789106 -3.52179219 -2.25158689

H 9.82474111 -5.00685745 -1.91641520

(2,2) [(UO_2_)_2_(OH)_2_(H_2_O)_6_] ^2+^

H 10.99715992 -3.26359536 -0.57942658

H 10.79223821 -3.76876013 0.88151632

H 8.25503097 -0.46445736 1.34088366

O 8.77253717 -0.97945184 0.69366538

H 2.94380965 0.41693143 -0.38172765

H 9.70123863 -0.94168851 0.99218025

O 2.91460321 -4.39143628 -1.94923799

U 7.84137283 -3.18185651 -0.21133689

U 4.13704100 -2.43908789 -0.95865936

O 5.74556598 -4.11327665 -0.67590499

H 8.43757789 -6.31841479 -0.31767023

O 1.82823908 -1.50607247 -1.57745735

O 10.37621491 -3.23934225 0.17419673

H 4.21519868 0.34488083 0.52353235

H 6.45512618 -0.81770664 -0.91688523

H 3.08758874 -4.72226644 -2.85113442

H 8.60030400 -5.79413537 -1.78267441

O 8.23618233 -2.75588830 -1.90366593

O 3.49715197 -2.91230636 0.64457832

H 5.48042375 -4.70706414 0.05507207

O 8.52745574 -5.50746239 -0.85245768

O 3.83001648 0.01383897 -0.30961186

O 4.59517214 -2.03563204 -2.64046230

O 6.18822434 -1.49926988 -0.26546877

H 2.48118568 -5.11909808 -1.46496515

O 7.60727418 -3.74551638 1.47143895

H 1.59015279 -1.35400927 -2.51251474

H 1.00396399 -1.78186566 -1.13227723

(3,4) [(UO_2_)_3_(OH)_4_(H_2_O)_7_]^2+^

H 11.06021427 -1.99433713 -0.50740235

H 11.07654116 -2.64054975 0.90796381

H 7.52795676 -0.24563985 1.29507239

O 2.81218814 -1.54919009 -0.39633630

O 1.98507425 -4.02701475 -1.29576682

H 9.09075925 -0.30096628 1.20819154

O -0.82165120 -3.61460504 -2.62405056

U 8.05429016 -2.96936058 -0.14810855

U 0.80029293 -2.04582128 -1.44669139

O 6.43023976 -4.52956501 -0.64783613

H 9.60166873 -5.76408587 -0.12539178

H 0.40098557 1.10025066 -1.41431412

O 10.49566574 -2.19272030 0.26409414

O 4.50424554 -2.82750992 -2.49968819

H 6.03723302 -1.18545151 -0.92951732

O 4.02248222 -3.95466356 0.87979709

U 4.28184144 -3.39864444 -0.81185251

H 1.67034032 -4.57025115 -0.54496327

O -0.01443712 -2.38457957 0.11663948

H 2.70076942 -1.44781398 0.57087023

O 9.53945347 -4.95895692 -0.67292163

O 0.97068457 0.46515847 -0.94118435

O 1.45896880 -1.74218378 -3.08428850

O 6.01814742 -1.85770005 -0.21804982

H -1.40093929 -4.25463430 -2.16979778

O 7.98720411 -3.55659122 1.54737077

H 9.69583281 -5.24241822 -1.59346986

O 8.34127510 -2.46437046 -1.84637069

H 1.87657527 0.82682890 -0.96593101

H 6.32528669 -5.14341126 0.10711797

O 8.27087949 -0.56951637 0.75206931

H -0.56447903 -4.03237294 -3.46764925

O -1.33075642 -0.67968974 -2.02419629

H -1.71441186 -0.68640095 -2.92167323

H -2.09071391 -0.71022682 -1.41212386

O 4.16469173 -5.78734926 -1.78605292

H 4.97555028 -6.07376810 -2.24777191

H 3.43409018 -5.90632285 -2.42230305

3.5: 3A [(UO_2_)_3_(OH)_5_(H_2_O)_4_]^+^

U 0.34578146 -2.77515099 1.06396866

H -0.28552204 -8.76274327 1.67394150

O -0.02815418 -0.92885751 0.07129759

O 6.67696180 -4.69013338 -0.09616665

H -2.49469829 -3.54262290 0.05825980

H -0.03776122 -8.72363056 3.21072790

H -2.79747518 -2.77868684 1.38161230

H -0.19181561 -0.11482765 0.58342375

U 2.08215936 -6.73348663 2.08919145

H 6.71439484 -5.39195240 -0.77216571

O 0.11268789 -1.98632896 2.68382355

O 3.26951600 -8.49794225 1.99535385

O 5.22418758 -1.65181116 -0.92381609

O 3.77980802 -4.25181969 -0.79923734

O 2.75021379 -2.35792491 1.24470994

O 5.54110708 -2.74848863 1.94247508

U 4.58128476 -3.51831102 0.62720652

H 5.60951778 -0.79401774 -0.66611446

O 2.20894797 -6.65562514 3.89910148

H 2.89255014 -1.78696131 2.02529559

O 0.53441234 -3.67360578 -0.49252388

O 0.01296724 -8.18465393 2.39976516

O 1.83773592 -6.76028893 0.29874006

O 4.02827707 -5.24392788 1.96108524

O -2.13239458 -3.33799944 0.94014987

H 0.67664048 -4.56957401 3.20695198

H 3.46010648 -8.91258693 1.13311017

O 0.76499714 -4.77670137 2.25529431

H 4.78009728 -1.51534927 -1.78161382

H 4.38746351 -5.17756304 2.86808447

H 7.51057748 -4.73556233 0.40751799

3.5: 3B [(UO_2_)_3_(OH)_5_(H_2_O)_5_]^+^

U 0.24572426 -2.99442342 1.12631321

H -0.52581614 -7.99868377 1.95719081

O 1.29152713 -1.08632161 0.60584862

O 6.88307344 -4.80431937 -0.01840264

H 2.63639284 -1.31851004 0.07518853

H 0.61786011 -8.74789680 2.71564265

H -2.81169669 -2.04747081 1.00537765

H 1.29286349 -0.36279433 1.26026751

U 2.14426082 -6.36538177 2.03936528

H 6.72943690 -5.61232093 -0.54612674

O -0.12267510 -2.34663018 2.77261752

O 2.87990552 -8.35900460 2.37183141

O 6.23965139 -2.20369931 -0.83784216

O 4.04131834 -4.46922050 -0.93296978

O 2.47709504 -3.70502645 1.68439334

O 5.27448054 -2.88544369 2.04326790

U 4.60566794 -3.67078476 0.56893059

H 6.32674231 -1.26669000 -0.58108101

O 2.31807457 -5.89949647 3.78236441

H 2.64788261 -3.45337185 2.61594577

O 0.53747104 -3.61584962 -0.53947506

O 0.14250317 -7.89382461 2.65990135

O 1.94301040 -6.66610338 0.27347586

O 4.48505773 -5.65460163 1.67925591

O -2.12688604 -2.47293086 0.45840638

H -0.31446359 -5.06601949 2.89482037

H 3.09846853 -8.95103477 1.62847331

O 0.00546341 -5.10289195 1.97117043

H 7.14686242 -2.54665973 -0.94378216

H 4.88922354 -5.65105563 2.56917357

O 3.58091646 -1.61356078 -0.30359597

H -2.50967956 -2.60245695 -0.42828054

H 7.45146576 -5.09234261 0.72104065

H 3.46089439 -1.64458399 -1.27187359

3.5: 3C [(UO_2_)_3_(OH)_5_(H_2_O)_5_]^+^

U 0.52904684 -2.84401312 1.07200058

H -0.41494640 -8.48371675 2.48671838

O -0.54786444 -1.12055793 0.33844260

O 6.82686640 -4.39921691 0.30454834

H 5.72523198 -0.76169908 0.48789517

H 0.04737370 -7.99486133 3.89321961

H -2.19982571 -3.25498522 2.52241824

H -0.79870046 -1.06051344 -0.60188061

U 2.00662621 -6.47473850 1.98055524

H 7.26055196 -4.64890941 -0.53058767

O 0.65084354 -2.16167526 2.74697994

H 2.62579525 -3.78863071 2.82506701

H 4.03143771 -8.60564563 3.11288071

O 4.21642530 -4.00119662 -1.15427276

O 2.73557337 -2.06362896 0.25069709

O 5.01259443 -3.07104795 2.22244545

U 4.43198751 -3.58979972 0.59054708

H 3.75449776 -9.14916261 1.67763838

O 2.40170189 -6.18395944 3.72149484

H 2.56987346 -1.94860778 -0.70514672

O 0.43676548 -3.60703673 -0.57054484

O 0.35460724 -8.14705907 2.98059197

O 1.58861669 -7.13586769 0.35347660

O 4.08219342 -5.91157733 0.92057145

O -2.00994955 -3.12369944 1.57463575

H -0.44749563 -5.55820006 1.03046405

O 2.49650514 -4.12125336 1.91499969

O 0.08842442 -5.24142261 1.78354431

O 3.37803568 -8.60084601 2.38952558

H 4.13326245 -6.36345065 0.05681032

O 5.77011013 -1.50768957 -0.13980210

H -2.22761350 -2.18588196 1.38631908

H 7.53236465 -4.16759331 0.93480148

H 5.60329862 -1.12010985 -1.01898707

3.5: 3D [(UO_2_)_3_(OH)_5_(H_2_O)_5_]^+^

H 11.23359219 -2.86869485 -0.63419364

H 11.13494044 -3.71177945 0.67034987

H 8.07860716 -0.51615963 1.18859042

O 2.66066943 -1.25013062 -0.38920540

O 2.37659958 -3.95250819 -1.20090537

H 9.55806709 -0.94328298 1.47476290

O -0.19075863 -4.32330966 -2.71632812

U 8.09621600 -3.21913077 -0.21996655

U 0.69243790 -2.18259131 -1.54005878

O 6.16998724 -4.39790974 -0.63412618

H 8.87341879 -6.28360862 -0.58342878

O -1.30927981 -1.74912992 -2.29110117

O 10.65545951 -3.05717419 0.12895500

O 4.58693393 -2.33836369 -2.33445175

H 6.47588603 -1.07503498 -0.92779261

O 4.10150597 -3.35074401 1.09645011

U 4.29911699 -2.83980016 -0.62788930

H 2.04012570 -4.49283205 -0.45856830

O 0.01517257 -2.61120667 0.08291496

H 2.40318043 -1.15543687 0.54969159

O 9.08069368 -5.43648382 -1.02035344

O 0.27169563 0.36494468 -1.26861072

O 1.46479535 -1.77616919 -3.12259199

O 6.37379734 -1.66738108 -0.15470647

H 0.44592017 -4.67740066 -3.36531966

O 8.01894244 -3.87316381 1.45380002

H -2.07758500 -1.85692429 -1.70026295

O 8.40523704 -2.68771851 -1.90960420

H 0.98492984 0.90525998 -1.65751203

H 5.98805517 -4.97872930 0.13190097

O 8.85023046 -0.97580412 0.80452202

H -0.91199359 -3.91999966 -3.24095310

H 9.02417906 -5.61288426 -1.97874944

H -0.48984328 0.44691348 -1.87851473

3.5: 3E [(UO_2_)_3_(OH)_5_(H_2_O)_6_]^+^

U 0.33615468 -3.10376397 1.22306085

H -0.70056066 -6.26810244 1.50970515

O -1.13930007 -1.71329308 0.52859340

O 7.05591127 -5.07457566 0.96588736

H 2.90066676 -1.38560678 0.92677406

H -0.60403858 -6.67765347 3.00420076

H -1.09419199 -2.61390493 4.05419410

H -1.07435782 -0.74104292 0.57473455

U 2.16417735 -6.49886678 1.76432199

H 7.16356789 -5.80638870 0.32922397

O 1.40850795 -1.75064886 1.84901281

O 6.39836288 -2.32761683 0.69394741

O 2.33896906 -8.99623347 1.84260053

O 4.92184985 -4.14241883 -1.04931371

O 2.43159928 -4.33665077 0.52287374

O 4.54064433 -3.49615921 2.48857373

U 4.81829129 -3.79838630 0.72333721

H 2.58455601 -9.54145747 2.61124037

O 2.72037354 -6.68320694 3.47345735

H 2.29964137 -4.61041078 -0.40872749

O -0.60892522 -4.56383280 0.64333953

O -0.30898584 -6.95144280 2.11393561

O 1.65510136 -6.86240940 0.06842008

O 4.37630067 -6.16776336 1.31333639

O -1.35690533 -2.86575038 3.14994814

H 1.73912807 -4.24208474 3.50293517

H 6.68536751 -1.85469949 1.49724925

O 1.11655422 -4.57627660 2.82733512

H 2.07288678 -9.59793734 1.12419617

H 4.86811807 -6.31038391 2.14657949

O 3.52339455 -1.57617890 0.17193781

H -2.02619659 -2.21200791 2.86974267

H 7.69706594 -4.38795163 0.69223999

H 4.16891820 -0.84461165 0.12970650

O 1.21014897 -2.49708729 -1.13996397

H 2.05885888 -2.00252735 -1.04359750

H 0.60301782 -1.92170203 -1.64151572

3.5: 3F [(UO_2_)_3_(OH)_5_(H_2_O)_6_]^+^

H 11.05304781 -3.79758829 -1.20741720

H 10.95759536 -4.66438730 0.07925494

H 8.90877660 -0.91552208 1.19542858

O 2.26707498 -1.06033642 -0.33010757

O 2.68885455 -3.74673349 -1.02599314

H 10.26978481 -1.67625138 1.08307282

O 0.45223251 -4.71811741 -2.82855055

U 8.01928107 -3.44217194 -0.30356458

U 0.74847175 -2.42123593 -1.64683646

O 5.85700621 -4.16921146 -0.44858434

H 8.01295899 -6.58241270 -0.79540928

O -1.22631692 -2.53766262 -2.65113369

O 10.58915056 -3.87077940 -0.35206447

O 4.64549115 -1.79866338 -2.06027724

H 6.84304645 -0.92187587 -0.64246345

O 4.07596518 -2.70823267 1.38122520

U 4.33671416 -2.26931125 -0.35280789

H 2.39931270 -4.33294705 -0.29971203

O -0.01453611 -2.93701202 -0.08816642

H 1.89221575 -1.12032061 0.57155908

O 8.38443696 -5.80453950 -1.25199560

O -0.43882694 -0.09945049 -1.73590332

O 1.58385951 -1.88661712 -3.15744849

O 6.71505629 -1.60517876 0.04613770

H 1.16951059 -4.91505657 -3.45962742

O 8.03435767 -4.13222321 1.36122549

H -1.99002248 -2.83615828 -2.12303823

O 8.21341457 -2.93093628 -2.01882792

H 0.04060176 0.54681146 -2.28711146

H 5.62441111 -4.70980847 0.33170290

O 9.44201133 -1.48129939 0.60514903

H -0.31047728 -4.41706810 -3.36813040

H 8.13345547 -5.90931106 -2.18965920

H -1.11382373 -0.50364433 -2.33043219

O 4.55884773 0.28957817 0.21234922

H 4.74168493 0.52926560 1.13963178

H 3.60133347 0.46594856 0.07136092

3.5: 3G [(UO_2_)_3_(OH)_5_(H_2_O)_6_]^+^

H 10.78731406 -4.25401510 -1.60448681

H 10.53038701 -5.30810383 -0.48143399

H 9.07108243 -1.32242974 1.45183292

O 2.13473011 -1.00265809 -0.67214732

O 2.56734850 -3.75865978 -0.78560037

H 10.17161328 -2.38995778 1.69317310

O 0.23958899 -4.99919413 -2.43944232

U 7.96562899 -3.40992467 -0.28756190

U 0.83287530 -2.60152871 -1.65532032

O 5.70112582 -4.08861397 -0.40333941

H 6.50759235 -5.27279866 -1.71794564

O 5.04837575 0.03500151 -0.05049515

O 10.28988735 -4.41276374 -0.78188592

O 4.72820844 -1.90097456 -2.07651293

H 6.59279072 -0.81210856 0.37298381

O 3.88220426 -2.32419900 1.41160160

U 4.36194848 -2.04342020 -0.31465317

H 2.37180298 -4.10310704 0.10820031

O -0.15075873 -2.81523602 -0.15808665

H 1.83807590 -0.74290313 0.22259682

O 7.46730814 -5.37773573 -1.93478059

O -0.07432156 -0.21400952 -2.12553821

O 1.66972225 -2.49837314 -3.24446627

O 6.86052515 -1.74894092 0.58194482

H -0.06498654 -5.65286651 -1.78230491

O 8.04474532 -4.49096932 1.16210163

H 5.20609936 0.56122865 -0.85570983

O 8.15683319 -2.49400760 -1.83052325

H 0.71282950 0.34139314 -1.95354087

H 5.52750295 -4.63182333 0.38963942

O 9.67738183 -1.93357219 0.98638691

H 0.99327937 -5.42348616 -2.89312672

H 7.54626691 -5.09875446 -2.86650557

H -0.36314757 -0.00650274 -3.03392596

O -1.46430524 -2.57144997 -2.92816475

H -2.27703213 -2.60069642 -2.38945290

H -1.60428137 -3.21431220 -3.64841982

3.5: 3H [(UO_2_)_3_(OH)_5_(H_2_O)_7_]^+^

U 0.21698085 -2.87213958 1.13529532

O 2.60157097 0.21332595 0.05249732

O 0.22414173 -1.01116675 -0.05431793

O 6.45517269 -5.11509493 0.81529551

H 3.61140759 -0.77329252 -0.53649059

H 2.56076149 1.10767417 -0.32767991

O 6.03375864 -7.43405896 1.80541093

H 0.09426088 -1.15242545 -1.01139536

U 1.97797094 -6.70591423 2.07786648

H 6.26871954 -6.07367271 1.17817861

O 0.06073503 -1.96618766 2.69486285

O 3.49220495 -8.24231492 1.62423807

O 6.64837388 -2.84045394 -0.76492081

O 3.86274383 -4.44208280 -0.73371786

O 2.53654451 -2.56288751 1.28423466

O 5.29154558 -2.57850799 1.99306480

U 4.55712023 -3.52026880 0.64465632

H 6.41480081 -2.05298437 -1.29485298

O 2.22085021 -6.80584147 3.87020893

H 2.71178421 -1.91540557 1.99522577

O 0.25069469 -3.82862199 -0.39412003

H 1.66218757 -0.15767816 0.01621142

O 1.57486884 -6.65215729 0.32142671

O 3.76584687 -5.16517056 2.07400737

H 5.08289968 -7.77457975 1.76238313

H 0.50920112 -4.62592999 3.35238125

H 3.45102479 -8.54120319 0.69549054

O 0.55447289 -4.84498407 2.40061729

H 7.01282406 -3.49974161 -1.38378086

H 4.07525019 -5.04808016 2.99311364

O 4.25141322 -1.51126935 -0.89123342

H 6.59002788 -8.15259585 1.45871122

H 7.19404680 -4.77214188 1.35068847

H 3.82322409 -1.82763785 -1.70868097

O 0.10439901 -8.38179497 2.36481733

H -0.02135730 -8.92211040 3.16562877

H -0.35381401 -8.84118867 1.63836605

O -2.30370003 -3.09445300 1.10689015

H -2.82646019 -3.42630203 0.35489898

H -2.90661386 -2.56329365 1.65791851

4.7: 4A [(UO_2_)_4_(OH)_7_(H_2_O)_5_]^+^

U 0.95997546 -2.26542785 -1.62948592

O -0.71243071 -0.56980938 -2.03743697

O -0.88463876 -3.22797577 -2.85227959

O 2.69815267 -1.26956967 -0.46118335

O 2.50355730 -3.96307800 -1.28109161

H -0.39841597 -0.01710024 -2.78178569

O -3.86006715 -3.86958925 -3.33465248

H -0.59902635 -3.31179881 -3.78434434

U -2.63359359 -1.74220457 -2.61136193

O 6.23829832 -4.37928073 -0.74523238

O 0.12761115 -2.67245290 -0.08388722

O -5.15083109 -1.16624868 -2.81513952

O 10.82788828 -2.99969867 0.52835417

O 4.77548629 -2.28592605 -2.36156306

H 6.65383661 -1.09975615 -0.91103686

O 4.21738591 -3.33608541 1.04748925

U 4.52925005 -2.82309344 -0.65923757

H 2.22556078 -4.52147013 -0.52753885

O -2.94425322 -2.31429213 -0.94221584

H 2.47179840 -1.19799970 0.48800551

O 1.70781312 -1.83752159 -3.21070994

O -3.20428892 0.64564529 -1.83679302

O -2.49290777 -1.24258441 -4.33179640

O 6.42808742 -1.61417439 -0.10921259

H -3.68321136 -4.66282337 -2.79318747

O 8.00610235 -3.95031330 1.51748879

H -5.82752831 -1.74030317 -2.40896889

O 8.62057530 -2.57464077 -1.77322953

H -4.06470786 0.83473456 -1.41789279

H 6.12514980 -5.03237975 -0.02577063

O 8.74057433 -1.05515776 1.12467620

H -3.84585371 -4.17526790 -4.26106977

H -5.51768482 -0.89208398 -3.67699250

H -2.51782116 0.98308438 -1.22958875

U 8.31616569 -3.31853175 -0.15015611

H 9.76191411 -5.18778493 -1.60592195

O 9.49079098 -5.06805379 -0.67665780

H 8.94224871 -1.22725009 2.06347079

H 11.01952486 -3.89765960 0.17250681

H 7.78311101 -0.82113847 1.09470675

H 11.32897067 -2.38277770 -0.03705529

4.7: 4B [(UO_2_)_4_(OH)_7_(H_2_O)_5_]^+^

U 0.32639545 -2.83136927 1.12990643

O 0.09536152 -0.62193923 -0.15216031

O -1.61065485 -2.46295905 1.95354138

O 9.93044871 -4.07856810 0.35963076

H 7.99296139 -1.13954916 -3.30331181

H 0.01622346 0.16095373 0.42420655

O 3.49068367 -8.71132724 1.53380939

H -2.38642334 -2.86057870 1.51578261

U 1.91798702 -6.72167574 1.95521240

O 6.98810117 -3.81723924 1.20728969

O 1.11470723 -1.84203020 2.42866128

O 0.30018602 -8.08177425 1.66753528

O 10.09759928 -2.06233636 -1.89606009

O 4.79597040 -4.81363809 -0.60416763

H 5.64617836 -1.13764175 -0.02417406

O 4.52639845 -2.51778711 2.16036489

U 4.60106109 -3.69258478 0.79072924

H 10.70357102 -1.36610597 -1.57963855

O 2.03415575 -7.06623031 3.73573287

H 2.25157548 -3.84598925 -0.64845320

O -0.38580276 -3.70359527 -0.29442098

H -0.62830625 -0.54707332 -0.80114509

O 1.96780772 -6.32345050 0.17975930

O 5.76908553 -1.98586790 -0.49629052

H 3.49792814 -9.08130357 0.63182416

O 8.45171929 -1.32359966 0.59798242

H -0.13224234 -8.16068437 0.79708149

O 7.70581546 -4.01883281 -1.64651042

H 10.66901161 -2.77491158 -2.23925816

H 7.14097984 -3.38895399 2.07318655

O 7.50285501 -1.03943461 -2.46582715

H 3.38284625 -9.47482435 2.13072882

H 10.38177373 -3.84795384 1.19331068

H 6.55315757 -1.08134655 -2.69103883

U 7.97798197 -2.65535517 -0.51437185

H 9.72945326 -5.03204528 0.42688937

O 2.46564880 -3.23490947 0.08747168

O 3.89884385 -5.36710532 2.20964833

O 0.67086924 -4.80043701 2.38754182

H 4.00467610 -5.17627862 3.16172783

H 0.59027252 -4.64149229 3.34713246

4.7: 4C [(UO_2_)_4_(OH)_7_(H_2_O)_6_]^+^

U 1.50217115 -0.64032158 -2.72410346

O 0.86239079 -1.46655044 -0.55530955

O 0.21842400 -2.77131186 -2.86681430

H 8.33046035 -3.88631070 2.32937921

H 5.57534294 -7.32472691 0.52746329

H 0.09906185 -0.98408211 -0.18418353

O -0.59948770 -5.62385324 -2.11243587

H -0.70646739 -2.52008969 -3.06000314

U 0.53178430 -3.71704836 -0.78881417

O 5.10487592 -3.11468930 -1.43473060

O 3.00855279 -1.70955098 -2.50098919

O 0.35192626 -5.78197323 0.75801923

O 6.29407337 -6.77716775 0.89774653

O 5.88368580 -0.36452148 -2.05031254

H 5.98925696 -1.76812186 1.62530951

O 3.53570962 -1.30471275 0.58281248

U 4.69785301 -0.76713560 -0.74354371

H 7.22028409 -4.56448212 3.18594354

O 2.07910735 -4.47140226 -1.39811499

H 7.12481157 -7.22460004 0.65161200

O 0.00654362 0.39066920 -2.72588019

O 1.87718093 -3.28451367 1.26256756

O -1.13160056 -3.36508296 -0.18438186

O 6.30573080 -2.09049128 0.75902633

H 2.79171412 1.58113289 -1.18210290

O 4.53217903 -4.30973118 1.33757318

H 0.47385366 -6.68678308 0.41603073

O 7.47351377 -4.49176710 -0.76349582

H 2.63009222 -3.89946373 1.43210227

H 5.57317695 -3.05195954 -2.28842598

O 7.57070347 -4.49609594 2.27864763

H -0.28647449 -5.68610436 -3.03547852

H -0.39079931 -5.83176545 1.38819921

H 2.36895231 -2.42143614 1.09106940

U 5.99624220 -4.26422530 0.24786651

H 3.62745930 -5.38674484 -1.27146487

O 4.46685596 -5.90155797 -1.11728937

O 2.95115286 0.70979489 -1.58581381

H 6.08010258 1.46316816 0.12754261

O 1.88985174 -0.19704318 -4.76421632

H 1.33142663 0.39224455 -5.30385967

O 5.35690517 0.88512420 0.43263891

H -1.57369259 -5.62764599 -2.16619957

H 4.79520174 -6.09521597 -2.01550849

4.7: 4D [(UO_2_)_4_(OH)_7_(H_2_O)_6_]^+^

U 0.94616524 -1.37987127 -1.27332751

O -1.50159721 -0.47448920 -1.97666712

O -0.29598460 -2.91253469 -2.74021962

O 3.12570734 -0.82259634 -0.25649040

O 2.61340713 -3.19007368 -1.69359275

H -1.23755223 0.07187234 -1.17924793

O -2.18171285 -4.79567937 -3.03269951

H 0.06524403 -2.76668622 -3.63676770

U -2.54843708 -2.21396917 -2.71653085

O 6.17114631 -4.33082923 -1.12124549

O 0.41306997 -2.42275846 0.10599248

O -4.84986097 -3.14141448 -3.45945381

O 10.59160895 -4.44808766 0.14288489

O 5.14725516 -1.58057949 -2.15096083

H 7.00907235 -1.18615981 -0.36493119

O 4.11496739 -3.51665560 0.72163278

U 4.55595429 -2.52508500 -0.72612902

H 2.20551616 -3.92795339 -1.19879568

O -2.98622619 -2.72930911 -1.04124451

H 3.35756366 -0.00537017 -0.74055611

O 1.55088080 -0.45868709 -2.70916582

O -4.22892241 -0.23116185 -2.71858512

O -2.35657275 -1.89874335 -4.48961605

O 6.74200581 -1.92533901 0.21881076

H -2.44448021 -5.29006886 -2.23367271

O 7.77490661 -4.77602752 1.15772858

H -5.45338666 -3.62120063 -2.86377555

O 8.83492463 -2.65140268 -1.53238191

H -3.60582307 0.50031968 -2.53231476

H 5.81171143 -5.03925404 -0.54905756

O 9.14326821 -2.14006794 1.63930526

H -1.21577706 -4.61692408 -2.91223029

H -4.94331165 -3.55612122 -4.33649463

H -4.62056966 -0.02069540 -3.58696496

U 8.19095998 -3.67734140 -0.20349456

H 7.65860922 -5.67719020 -2.30603564

O 8.53199136 -5.62445129 -1.86593533

H 9.41668054 -2.54566981 2.48381483

H 10.88315072 -5.21100028 0.67500494

H 8.37860163 -1.56950593 1.85994705

H 11.39174893 -4.05329242 -0.24965495

O 0.16234274 0.38084054 -0.17780802

H -0.07984631 0.23004591 0.75441333

H 9.17822079 -5.50480905 -2.58739551

4.7: 4E [(UO_2_)_4_(OH)_7_(H_2_O)_7_]^+^

U 0.98822655 -1.87130624 -1.45538449

O -0.92716308 -0.40631005 -2.07276377

O -0.74154159 -3.11719108 -2.64137786

O 3.04456598 -1.51637860 -0.28701703

O 2.10664912 -3.90206963 -1.42344380

H -0.60333282 0.15647344 -2.80440195

O -3.57650876 -4.14883428 -3.20961353

H -0.37897817 -3.28327388 -3.53413913

U -2.62470385 -1.81715960 -2.65589962

O 6.53416672 -4.54266943 -0.88520395

O 0.19465348 -2.20831798 0.12817181

O -5.20256022 -1.61103662 -3.05358704

O 10.82695911 -3.51549289 0.42893764

O 4.79633522 -2.57581640 -2.47441769

H 6.24560321 -1.15493800 -0.57022674

O 4.16204142 -4.10677351 0.73436738

U 4.51843406 -3.33130551 -0.86034355

H 1.79173932 -4.43635079 -0.66711170

O -2.97167622 -2.25438472 -0.94888836

H 2.88284287 -1.62291383 0.67178576

O 1.73637081 -1.50742845 -3.05246117

O -3.59278111 0.53333433 -2.19444392

O -2.44464410 -1.47804380 -4.41495936

O 6.15791391 -1.92444941 0.02599387

H -3.28526090 -4.84803176 -2.59313438

O 8.00397713 -3.92593633 1.54882921

H -5.80050640 -2.26089471 -2.63823315

O 8.57460583 -2.15499702 -1.56048279

H -4.52025802 0.60077946 -1.89932958

H 6.43994972 -5.19468414 -0.16164973

O 9.33504736 -1.38244701 1.06781054

H -3.45989304 -4.52506289 -4.10220731

H -5.52676759 -1.50340708 -3.96769546

H -3.05750528 1.01438044 -1.53528421

U 8.28491756 -2.98892866 0.02308364

H 8.17056867 -5.25539895 -1.62891021

O 9.09792029 -5.11501357 -1.31905687

H 9.61992955 -0.60387098 0.55493876

H 10.82336323 -2.69997970 0.98695129

H 4.50018806 -5.52472814 -3.10054375

H 10.94742514 -4.25743922 1.05035898

O 1.48244611 0.63390926 -0.84862076

H 0.95629694 1.04182623 -0.13653737

H 9.60201300 -4.83790785 -2.10654536

O 4.04649515 -5.51615016 -2.23771165

H 3.11542564 -5.24670728 -2.41867679

H 2.36924746 0.45859034 -0.46319862

4.7: 4F [(UO_2_)_4_(OH)_7_(H_2_O)_7_]^+^

U 1.11072146 -1.95424437 -1.40778899

O -1.66877793 0.17106634 -3.00086435

O -0.72415912 -2.73183514 -2.73822384

O 3.17522614 -1.69883806 -0.17011815

O 2.37733512 -4.00948509 -1.57333745

H -0.99016198 0.37551237 -2.29305575

O -2.87693092 -4.14765931 -2.05289524

H -0.44479528 -2.74946737 -3.67448627

U -2.84729467 -1.56946103 -2.68797656

O 6.79362574 -4.52554856 -0.91356211

O 0.34231112 -2.65048563 0.08578856

O -5.34074581 -2.30379799 -2.47491450

O 10.79276928 -2.50258807 0.65036261

O 4.92121544 -2.52399396 -2.40181178

H 6.33107413 -1.20616757 -0.42540774

O 4.31129567 -4.31217898 0.66943744

U 4.58449377 -3.39713101 -0.86377713

H 1.99676846 -4.58662626 -0.88125540

O -2.80799099 -1.35805528 -0.88744258

H 2.96594040 -1.77668368 0.78131666

O 1.90981459 -1.38347146 -2.92726896

O -4.34036812 0.53962015 -3.05382291

O -3.07809226 -1.96705152 -4.44264315

O 6.34250973 -2.01264356 0.12876264

H -2.91659752 -4.23961611 -1.08212148

O 8.27263658 -4.10789469 1.47298369

H -5.76680920 -2.38950862 -1.60290953

O 8.68574378 -2.19716607 -1.53993894

H -3.51864460 1.06098168 -3.20802343

H 6.64637181 -5.31095537 -0.35033812

O 8.54447043 -1.02547437 1.48116363

H -1.90619458 -4.11971840 -2.25352017

H -5.62243637 -3.08282007 -2.98867968

H -4.83005581 0.56199707 -3.89662863

U 8.34515193 -3.11913366 -0.03099661

H 9.82279600 -5.83923673 -0.77000436

O 9.90698838 -4.90240503 -1.02788484

H 9.23935297 -1.00093395 2.16531251

H 11.36841701 -3.17418031 1.06160110

H 7.69817764 -0.85425601 1.93694759

H 11.34757497 -2.05501130 -0.01601718

O 0.18052166 -0.00619308 -1.00750168

H -0.37602401 0.01667789 -0.20534139

H 9.99843666 -4.90416355 -1.99950678

O 4.44386389 -5.53397053 -2.36606222

H 3.51725774 -5.34610381 -2.63923600

H 4.98215377 -5.47365120 -3.17715628

4.7: 4G [(UO_2_)_4_(OH)_7_(H_2_O)_8_]^+^

U 1.05184913 -1.36989675 -1.07581100

O -1.21353381 -1.73263409 -0.47383512

O -0.12219827 -1.70244561 -3.06814573

H 9.43272904 -1.09871550 -0.75282545

H 11.13957350 -5.27039346 0.17647430

H -1.64368083 -0.86123181 -0.35595665

O -3.02185853 -3.54125225 -4.28115201

H -0.33606630 -0.84545472 -3.48834072

U -2.20329727 -2.71335311 -2.42705482

O 6.36568123 -3.83636076 0.64867030

O 1.45115020 -3.14102065 -0.90407827

O -4.88735609 -5.25449472 -3.36027536

O 11.23331611 -4.36558433 -0.19006309

O 4.86170063 -2.57404217 -1.76144619

H 7.44630243 -1.93157681 -1.79712002

O 5.60521234 -1.21023266 1.51776336

U 5.26942508 -1.94244683 -0.09051870

H 10.76805363 -1.45181231 -0.02520287

O -1.33325871 -4.24388333 -2.03024014

H 11.57824438 -4.48665703 -1.09455440

O 0.71067765 0.39394008 -1.18440793

O -4.22955418 -3.74331901 -1.34908029

O -3.00872075 -1.12663866 -2.75779850

O 7.45017128 -1.70565006 -0.84441518

H 3.14640895 -0.02363381 0.37833224

O 9.00066334 -3.13839254 1.65505290

H -4.63587154 -6.19461273 -3.34182441

O 8.40928764 -4.30512169 -1.71780451

H -4.58139332 -4.42158816 -2.03822350

H 6.43673694 -3.82282762 1.62461187

O 10.17541155 -1.74193740 -0.74422805

H -3.49024497 -2.91996540 -4.87096869

H -4.18039324 -4.79674261 -3.90146694

H -4.05671287 -4.26545269 -0.54415108

U 8.69621308 -3.78420962 -0.00734530

H 8.79256304 -6.56561503 0.03803105

O 8.97845920 -5.81927348 0.63778673

O 3.12287384 -0.88583162 -0.08126442

O 3.54592876 -3.64582510 0.75192048

O 2.76082792 -1.36411653 -2.97441469

O 0.73962042 -1.41205683 1.49880771

O 5.41288238 0.44611789 -1.13579866

H 6.31419245 0.72504760 -1.38408306

H 4.84906737 0.64249211 -1.90769400

H 1.41603445 -1.89433528 2.01157034

H -0.10594298 -1.87718761 1.65623497

H 3.95721047 -4.53135835 0.72936477

H 2.76321602 -3.68158983 0.13778073

H 2.37308403 -1.79202990 -3.76201405

H 3.54974358 -1.91506317 -2.71332194

4.7: 4H [(UO_2_)_4_(OH)_7_(H_2_O)_8_]^+^

U 1.33995496 -1.87488400 -1.91815018

O -0.86313474 -1.28383128 -0.77144474

O -0.61086776 -3.25087578 -2.77128739

H 7.56608274 -2.39362719 2.90772882

H 10.39269378 -5.83310531 1.89853634

H -0.98565062 -0.38015051 -1.12511316

O 2.37491077 -3.16914641 -3.44076278

H -0.24313082 -4.13349718 -2.56414968

U -2.46176634 -2.69852865 -1.60665437

O 7.21780960 -3.39348193 -1.29438140

O 1.58178076 -3.23208181 -0.75148037

O -4.96142202 -3.26209569 -1.38993848

O 10.33639210 -4.86085897 1.86675453

O 6.31535041 -0.35035644 -1.55609320

H 7.68588288 -0.98616468 0.84134662

O 4.51789656 -3.00619311 0.11798069

U 5.37292485 -1.66256376 -0.72937067

H 7.99160778 -3.70855907 3.64493513

O -2.16545387 -4.15238673 -0.58073709

H 11.25468415 -4.54022753 1.80437528

O 0.91097477 -0.53906886 -3.06957085

O -3.20020164 -1.61917995 0.65294767

O -3.02963243 -1.29537440 -2.58920194

O 7.16017870 -1.80195498 0.95435176

H 3.59375232 -0.17203606 -2.46799209

O 7.43984977 -5.05829626 0.98111457

H -5.32622544 -4.01737568 -0.89395379

O 9.77997229 -2.47385368 0.09562446

H -3.28835114 -2.25348469 1.38960416

H 6.59259421 -4.07987872 -1.60673789

O 8.31423675 -3.01835840 3.03628803

H 2.32422860 -2.83047620 -4.35406164

H -5.67235167 -2.59817872 -1.44984279

H -2.32653366 -1.19070510 0.79358112

U 8.47778537 -3.65773460 0.51241404

H 4.61695028 0.70651825 0.57322338

O 4.37660330 -0.23271992 0.67529749

O 3.49244474 -0.98400048 -1.94059686

O 4.95271329 -3.27158595 -2.80984847

O -3.09702835 -4.10818145 -3.70007551

O 1.77380128 -0.38144284 0.15127705

O 9.74857643 -5.18902742 -1.13576185

H 9.23795304 -5.85921000 -1.62724096

H 10.23718571 -4.68735959 -1.81566048

H 2.76859208 -0.31338008 0.38584049

H 1.35043317 -0.74959641 0.94683369

H 5.42538359 -2.97888625 -3.60956736

H 3.96006444 -3.27396463 -3.07545844

H -3.79018712 -3.74549871 -4.28298103

H -2.26306018 -4.03274985 -4.20812941
